# Diagnostic and Management Applications of ChatGPT in Structured Otolaryngology Clinical Scenarios

**DOI:** 10.1002/oto2.67

**Published:** 2023-08-22

**Authors:** Roy W. Qu, Uneeb Qureshi, Garrett Petersen, Steve C. Lee

**Affiliations:** ^1^ Department of Otolaryngology–Head and Neck Surgery Loma Linda University Medical Center Loma Linda California USA; ^2^ Department of Otolaryngology–Head and Neck Surgery Loma Linda School of Medicine, Loma Linda University Medical Center Loma Linda California USA

**Keywords:** artificial intelligence, ChatGPT, large language model, otolaryngology

## Abstract

**Objective:**

To evaluate the clinical applications and limitations of chat generative pretrained transformer (ChatGPT) in otolaryngology.

**Study Design:**

Cross‐sectional survey.

**Setting:**

Tertiary academic center.

**Methods:**

ChatGPT 4.0 was queried for diagnoses and management plans for 20 physician‐written clinical vignettes in otolaryngology. Attending physicians were then asked to rate the difficulty of the clinical vignettes and agreement with the differential diagnoses and management plans of ChatGPT responses on a 5‐point Likert scale. Summary statistics were calculated. Univariate ordinal regression was then performed between vignette difficulty and quality of the diagnoses and management plans.

**Results:**

Eleven attending physicians completed the survey (61% response rate). Overall, vignettes were rated as very easy to neutral difficulty (range of median score: 1.00‐4.00; overall median 2.00). There was a high agreement with the differential diagnosis provided by ChatGPT (range of median score: 3.00‐5.00; overall median: 5.00). There was also high agreement with treatment plans (range of median score: 3.00‐5.00; overall median: 5.00). There was no association between vignette difficulty and agreement with differential diagnosis or treatment. Lower diagnosis scores had greater odds of having lower treatment scores.

**Conclusion:**

Generative artificial intelligence models like ChatGPT are being rapidly adopted in medicine. Performance with curated, easy‐to‐moderate difficulty otolaryngology scenarios indicate high agreement with physicians for diagnosis and management. However, a decreased quality in diagnosis is associated with decreased quality in management. Further research is necessary on ChatGPT's ability to handle unstructured clinical information.

Artificial intelligence (AI) has rapidly made inroads across many industries with health care being no exception with applications ranging from diagnosis to drug development and treatment plans. Large language models (LLMs), colloquially known as chatbots, utilize natural language processing to generate human‐like conversations and have been ubiquitously described in popular media.^1^ In medicine, it is crucial we understand this technology as a tool used by clinicians as well as by the public. As one of the most advanced and publicly available chatbots, chat generative pretrained transformer (ChatGPT) was mainly trained using publicly available data until September 2021 and has been shown to have high accuracy in generating human‐like responses to a wide range of questions in seconds.^2^ Within medicine, recent studies have shown that ChatGPT can pass the United States Medical Licensing Exam.^3^ However, it is unclear how well ChatGPT can provide a diagnosis and treatment plan when provided in an open‐ended clinical situation. Similarly, little is known about how its responses compare with human evaluation.

Early evidence demonstrates a mixed picture of the role of AI in diagnostics. One study found that AI models had sufficient accuracy in diagnosing and providing treatment plans and suggested the possibility of using AI as a tool in health care.^4^ While another study compared the diagnostic accuracy of an AI model with that of internal medicine physicians in diagnosing and treating common chief complaints and found that the AI model had a significantly lower diagnostic and treatment accuracy than human physicians.^5^

With public interest at a high, ChatGPT may become an increasingly utilized tool for both patients and clinicians. Its efficacy in diagnosis and treatment for specialty care, such as otolaryngology, has not been well evaluated. In this study, we compare the diagnostic accuracy and the treatment plans of ChatGPT with that of human physicians using a series of otolaryngology clinical vignettes. The results of this study highlight a relatively high accuracy in both diagnosis and treatment for common otolaryngology pathology but suggest that treatment accuracy decreases when a poor list of differential diagnoses is generated.

## Methods

### Institutional Review Board (IRB) Determination

This study was exempt from review by the Loma Linda University Health IRB as it does not constitute human subject research.

### ChatGPT Interface and Survey Generation

Twenty clinical vignettes were conceptualized and prepared by the authors of this paper. These represented clinical presentations across multiple specialties within otolaryngology with the intent of having varying degrees of difficulty. While these were uniquely created for this study primarily using clinical experience, materials such as otolaryngology textbooks and question banks were also utilized. The clinical presentations were designed to be similar to the type that would be encountered during in‐service tests and board examinations. These vignettes were then fed into ChatGPT 4.0 from April 26, 2023 to April 27, 2023 in 2 stages. First, we provided the prompt in the following format and asked for differential diagnoses:

For the following scenario, give me 5 differential diagnoses in order of likelihood using primary scientific literature only. List references you used for each diagnosis. [Prompt]

After ChatGPT provided an answer, we next asked it to provide a treatment plan:

What is your treatment plan for your most likely diagnosis? Please include any consults, laboratory or radiographic studies, and treatments (medical and/or surgical) in your response

Example queries and outputs are provided in Supplemental Figure S1, available online. We collated the ChatGPT responses and created a survey asking participants to rate the difficulty of the prompt and report how much they agreed with ChatGPT outputs on a 5‐point Likert scale. The scale for difficulty is as follows: very easy (1), somewhat easy (2), neutral (3), somewhat difficult (4), and very difficult (5). The scale for agreement is as follows: strongly disagree (1), somewhat disagree (2), neutral (3), somewhat agree (4), and strongly agree (5). We distributed the survey to all attending physicians at our tertiary academic center via Research Electronic Data Capture, a secure web‐based application for surveys and databases. Full ChatGPT outputs and survey are also provided in Supplemental Figure S1, available online. ChatGPT 3.5 was utilized to begin writing the introduction of this paper. The original output for the introduction is provided in Supplemental Figure S2, available online.

### Statistical Analysis

Data were analyzed using IBM SPSS Statistics Version 27. Summary statistics are reported for vignette difficulty, diagnostic accuracy, and treatment plan for each item and overall: median, interquartile range, and range. Univariate ordinal regression was then performed examining the relationship between vignette difficulty and diagnostic and treatment accuracy. Odds ratios with a 95% confidence interval are reported. Statistical significance was determined at *p* < .05.

## Results

Eleven attending physicians provided complete responses to our survey, which represents a response rate of 61%. The specialty representation included 5 head and neck surgeons, 1 facial plastics surgeon, 1 laryngologist, 1 neuro‐otologist, 1 rhinologist, and 2 pediatric otolaryngologists (data not shown). Table 1 illustrates twenty clinical vignettes with the median difficulty rating, interquartile range (IQR), and full range. The lowest median score was 1.00 for prompts #3, #9, #15, and #17. The highest median score was 4.00 prompt #5. The spread varied for each vignette with IQR ranging from 0 to 2.00. Overall, the median difficulty was 2.00 with an IQR of 1.00. This suggests that attending physicians typically viewed the vignettes as easy but there was modest variation.

**Table 1:** Clinical Vignettes and Physician‐Rated Difficulty of Prompt

Prompt	Median (IQR)	Range
1. A 25 year‐old male was punched in his right eye while intoxicated at a bar. He reports right eye pain and double vision when looking up but denies vision loss. On physical exam, his right eye is soft and does not exhibit proptosis, but there is superior gaze restriction.	2.00 (1.00)	1.00‐3.00
2. A 21 year‐old female presents with bilateral facial pain for 2 weeks. She states it started with a runny nose, cough and facial pressure 2 weeks ago. Her symptoms started to spontaneously improve after 7 days while taking Motrin. She started to develop worsening bilateral facial pain, particularly on her cheeks and forehead, associated with a fever and purulent nasal drainage. On physical exam, her frontal and maxillary sinuses are tender to palpation. There is green mucopurulent nasal drainage bilaterally, and there is edematous and erythematous mucosa throughout her nasal cavity.	2.00 (1.00)	1.00‐3.00
3. A 65 year‐old male with a 75 pack‐year tobacco smoking history presents with a 5 cm, painful, ulcerative, and raised lateral tongue mass. He says it has continued to grow for the past 3 months and sometimes bleeds. On physical exam, the mass is friable, fixed and is isolated to the lateral ventral tongue. There are no palpable cervical lymph nodes.	1.00 (1.00)	1.00‐3.00
4. A 33 year‐old obese female with a 3 year history of bifrontal headache. These headaches are worse in the morning and when she lays down. She presents for evaluation of intermittent rhinorrhea for 3 months. It is clear, only comes from the left side, and does not have a particular taste. She has mild bilateral nasal congestion but denies facial pressure and change or loss of smell. She has tried intranasal steroid and anticholinergic nasal sprays which have not improved her symptoms. She was in a minor car accident 6 months ago, but the airbags were not deployed and she did not hit her head. On physical exam, there is no active nasal drainage in a head neutral or chin tucked position. Her nasal mucosa is not erythematous and there is no turbinate hypertrophy.	2.00 (1.00)	1.00‐4.00
5. A 22 year‐old male presents with left‐sided facial pain for 2 days. He has been feeling abnormally fatigued for the past 3 months, has had 20 lbs of unintended weight loss, and has been soaking through his sheets overnight. He has also noticed some bumps around his waist while putting on his belt. The facial pain is associated with mild chunky nasal drainage. He otherwise denies facial numbness and vision changes. On physical exam, he has a low grade fever of 99F. His facial sensation, extraocular movements, and visual acuity are intact. His conjunctiva is also clear. His left nasal cavity has some mild crusting and mucinous nasal drainage. The head of the left inferior turbinate head appears dusky, as does a 1 cm area of his left hard palate. He has reports normal sensation in these areas but these areas do not bleed when pricked. There is nontender inguinal lymphadenopathy.	4.00 (1.00)	2.00‐5.00
6. A 40 year‐old female comes in with a 12 month history of progressive right hearing loss. It is associated with a high frequency non‐pulsatile tinnitus and intermittent vertigo. She denies otalgia and otorrhea. Recently, she has noticed the right‐side of her face drooping over the past 3 months. On physical exam, her external auditory canals and tympanic membranes are clear. There is no middle ear effusion. Her tuning fork exam lateralizes to the left, and air conduction is greater than bone conduction bilaterally. She has a House‐Brackmann grade 3 right facial paralysis.	2.00 (2.00)	2.00‐5.00
7. A 34 year‐old male with a history of allergic rhinitis, eustachian tube dysfunction, and recurrent episodes of right acute otitis media presents with 6 months of progressive right sided hearing loss and clear otorrhea. He denies otalgia, vertigo, and tinnitus. On physical exam, the right external auditory canal is clear. The right tympanic membrane has a superior retraction pocket with a possible tympanic membrane perforation, erosion of the malleus, and a small serous middle ear effusion. There is also a round, pearlescent mass in the right middle ear. His left external auditory canal and tympanic membrane are clear. His tuning fork exam lateralizes to the right, and air conduction is greater than bone conduction on the left but bone conduction is greater than air conduction on the right. His facial nerve is intact.	2.00 (1.00)	1.00‐4.00
8. A 34 year‐old male with no past medical history presents with left‐sided neck mass for the past year. It has been slowly growing for the past year and is nontender. During the same time, he has noticed episodes of spontaneous palpitations and sweating. During a visit with his primary care doctor last month, his blood pressure was noted to be 190/110s, and he was sent to the emergency room. On physical exam, there is a firm 3 cm mobile left level 2 neck mass. There are no other palpable masses, including thyroid masses. His oral cavity and oropharynx exam are normal and his vital signs are normal.	2.00 (1.00)	1.00‐4.00
9. A 25 year‐old female presents with left otalgia 4 hours after a boxing match where she sustained a strike to the left side of her head. She states it has become swollen and increasingly swollen. On physical exam, the helix and antihelix of the left pinna are swollen, fluctuant and have mild ecchymosis. The external auditory canal and tympanic membrane are clear bilaterally.	1.00 (0)	1.00‐2.00
10. A 29 year‐old male presents with 2 day history of right hearing loss. He was working at a coffee shop and noticed right‐sided hearing loss when he went home 2 days ago. He was not doing anything out of the ordinary that day. He recently came back from a trip from Colorado 1 week ago where he spent most of his time skiing. He has no other complaints and has not had hearing problems prior to 2 days ago. On physical exam, his pinna, external auditory canal, and tympanic membrane are normal. He brings an audiogram obtained yesterday that shows a 30 dB hearing loss from 250‐3000 Hz and a downsloping 30‐60 dB hearing loss from 4000‐8000 Hz in the right ear. Air and bone conduction thresholds are equivalent. Hearing in the left ear is normal.	2.00 (1.00)	1.00‐4.00
11. A 37 year‐old female presents with progressive shortness of breath. 5 months ago, she was in a housefire where she sustained severe inhalational injuries and was intubated for 14 days. After that incident, she has had progressive shortness of breath and has recently developed noisy, high‐pitched breathing over the past 3 weeks that is worse when she exercises. On physical exam, her voice is hoarse and weak. She has mild suprasternal retractions, biphasic stridor, and requires 2 L/min of supplemental oxygen to maintain oxygen saturations above 92%.	2.00 (1.00)	1.00‐4.00
12. A 39 year‐old female presents with a history of recurrent bilateral cheek swelling. These episodes are accompanied by intermittent low grade fever, up to 100F. She has had these symptoms for the past 2 months. She has also had to drink more water during these episodes, particularly when she eats. She is currently undergoing a work up for shortness of breath and pulmonary hilar fullness discovered on a chest X‐ray 3 months ago. Sometimes she has vision changes and facial weakness with these episodes, but these are rare. She states she is currently feeling normal. On physical, she is afebrile. Her parotid and submandibular glands are normal to palpable, and there is no palpable cervical lymphadenopathy.	2.00 (2.00)	1.00‐5.00
13. A 60 year‐old female with a history of Hashimoto's thyroiditis presents with a 1 year history of an enlarging neck. She has noticed the left side of her neck become more prominent, which bothers her, and has found it more difficult to swallow solids in the past 3 months. She has not had unintended weight loss. On physical exam, she has a 6 cm nodular neck mass, just left of midline, that moves with swallowing. There is no palpable lymphadenopathy, and her voice is normal.	2.00 (1.00)	1.00‐4.00
14. A 4 year‐old male presents with double vision for 1 day. His older brother developed a cough and nasal congestion 2 weeks ago which improved without intervention after 5 days. One week ago, the patient developed similar symptoms and nasal congestion. He woke up with a headache and forehead swelling this morning. On physical exam, he is febrile to 101.2F. His forehead is tense, swollen, tender and mildly fluctuant. His right eye has mild conjunctival injection, has mild proptosis. There is mild right‐sided abduction with lateral gaze, but visual acuity is intact. There is mild clear bilateral mucinous rhinorrhea. There is no neck stiffness.	2.00 (0)	1.00‐4.00
15. A 13 year‐old male presents with a 7 day history of sore throat. It is associated with odynophagia, poor oral intake, and a fever up to 101.4F. He denies cough. This has happened multiple times in the past, all of which have resolved with antibiotics. This time, however, his symptoms have not improved despite starting a course of amoxicillin/clavulanic acid 4 days ago. On physical exam, he is febrile, tachycardic, and has dry mucous membranes. His tonsils are enlarged and erythematous. There is significant leftward uvular deviation, and his voice is muffled. There is bilateral level 2 tender cervical adenopathy, right more than the left.	1.00 (1.00)	1.00‐3.00
16. A 42 year‐old female presents with a 1 year history of a hoarse and strained voice. She reports a 2 week history of cough and sore throat during the winter last year after which her voice became hoarse and strained. It waxes and wanes but is rarely normal. She also reports clearing her throat more frequently. She is a teacher and finds it difficult to teach because of the strain in her voice. Sometimes she also has difficulty yelling at her kids from across the house. On physical exam, her voice is hoarse and mildly strained. She has equal difficulty saying either sentence: “A dog dug a new bone” and “Harry is happy because he has a new horse.” There are no breaks in her voice, and there is no stridor.	3.00 (2.00)	1.00‐4.00
17. A 55 year‐old male underwent a coronary artery bypass graft 5 days ago and presents with a hoarse voice. He has had difficulty with eating, particularly with thin liquids. His voice has not improved since surgery. On physical exam, his voice is hoarse and breathy. When he takes a drink of water, he has overt signs of aspiration. He finds it easier to drink water when he turns his head to the left and tucking his chin.	1.00 (1.00)	1.00‐4.00
18. A 3 week‐old male presents with a 1 week history of left neck mass. Prenatal history is significant for gestational diabetes. The patient was born post‐term via forceps‐assisted vaginal delivery. The neck mass has been firm and slowly progressive in size. The patient has otherwise been doing well and has been feeding and gaining weight appropriately. On physical exam, the patient's head is turned slightly to the right. There is a 4 ×3 cm dense mass on the left neck that moves with head turning.	2.00 (2.00)	1.00‐4.00
19. A 10 year‐old female presents with a painful neck mass over the past 3 days. She states this has happened once before and resolved after a course of oral antibiotics. It is associated with a fever, swelling over the affected area, and pain with swallowing. On physical exam, there is a 3 cm midline tender neck mass that moves when swallowing. It is firm and partially fixed to the overlying skin which has mild erythema. There is bilateral tender cervical lymphadenopathy.	2.00 (1.00)	1.00‐4.00
20. A 41 year‐old female presents with 1 week history of left otalgia. It has been progressively worse since it started and has spread to her left eye today. She also endorses double vision and hearing loss but denies tinnitus, otorrhea and vertigo. She has insulin‐dependent diabetes mellitus. On physical exam, she appears fatigued. She has a mild left eye abduction deficit. She has normal pinna and external auditory canals bilaterally. There is a milky left middle ear effusion, but her tympanic membrane appears relatively normal. She has a temperature of 101.4F. His tuning fork exam lateralizes to the left, and air conduction is greater than bone conduction on the right but bone conduction is greater than air conduction on the left. Her facial nerve is intact.	2.00 (2.00)	1.00‐4.00
Overall	2.00 (1.00)	1.00‐5.00

Abbreviation: IQR, interquartile range.

Table 2 displays the 5 differential diagnoses provided by ChatGPT for each clinical vignette and the attending physician's agreement with the provided differential diagnoses. There was high agreement across all vignettes with most achieving a median score of 5.00. The lowest median score was 3.00 for prompt #19. The IQR for each prompt was small, usually 1.00 or less. Prompt #19 had the highest spread with an IQR of 2.00. Overall, there was again high agreement across all the vignettes with a median of 5.00 and IQR of 1.00.

**Table 2:** ChatGPT‐Provided Differential Diagnoses and Physician Agreement

Prompt	Differential diagnoses	Median (IQR)	Range
1	1. Orbital blowout fracture 2. Orbital contusion 3. Superior rectus/levator palpebrae superioris muscle injury 4. Cranial nerve palsy (specifically, oculomotor nerve palsy) 5. Periorbital hematoma	5.00 (1.00)	4.00‐5.00
2	1. Acute bacterial sinusitis 2. Acute viral rhinosinusitis 3. Allergic rhinitis 4. Fungal sinusitis 5. Dental infection with sinus involvement	5.00 (1.00)	4.00‐5.00
3	1. Squamous cell carcinoma (SCC) 2. Verrucous carcinoma: A rare, well‐differentiated variant of SCC 3. Oral pyogenic granuloma 4. Major aphthous ulcer (Sutton's disease) 5. Oral lichen planus	5.00 (0)	4.00‐5.00
4	1. Spontaneous cerebrospinal fluid leak 2. Idiopathic intracranial hypertension (pseudotumor cerebri) 3. Chronic tension‐type headache 4. Migraine without aura 5. Sinus headache (noninfectious)	5.00 (1.00)	2.00‐5.00
5	1. Granulomatosis with polyangiitis (formerly known as Wegener's granulomatosis) 2. Extranodal natural killer/T‐cell lymphoma, nasal type 3. Invasive fungal sinusitis (eg, mucormycosis or aspergillosis) 4. Tuberculosis infection with sinonasal involvement 5. Syphilis with sinonasal manifestations	4.00 (3.00)	1.00‐5.00
6	1. Vestibular schwannoma (acoustic neuroma) 2. Ramsay Hunt syndrome (herpes zoster oticus) 3. Ménière's disease 4. Temporal bone fracture 5. Autoimmune inner ear disease	5.00 (1.00)	4.00‐5.00
7	1. Chronic otitis media with cholesteatoma 2. Chronic serous otitis media 3. Adhesive otitis media 4. Tympanosclerosis 5. Congenital cholesteatoma	5.00 (0)	5.00‐5.00
8	1. Pheochromocytoma with a cervical paraganglioma 2. Cervical lymphadenopathy due to metastatic malignancy 3. Schwannoma of the cervical sympathetic chain 4. Branchial cleft cyst 5. Tuberculous lymphadenitis	5.00 (1.00)	4.00‐5.00
9	1. Auricular hematoma 2. Perichondritis 3. Traumatic ear laceration 4. Acute otitis externa 5. Temporomandibular joint dysfunction	5.00 (0)	5.00‐5.00
10	1. Sudden sensorineural hearing loss 2. Acoustic trauma 3. Barotrauma 4. Eustachian tube dysfunction 5. Meniere's disease (early stage)	5.00 (0)	5.00‐5.00
11	1. Subglottic stenosis 2. Tracheomalacia 3. Vocal cord dysfunction 4. Bronchiolitis obliterans 5. Reactive airway dysfunction syndrome	4.00 (1.00)	3.00‐5.00
12	1. Sjögren's syndrome 2. Sarcoidosis 3. Mikulicz's disease (IgG4‐related disease) 4. Recurrent parotitis 5. Lymphoma	4.00 (1.00)	1.00‐5.00
13	1. Goiter associated with Hashimoto's thyroiditis 2. Thyroid nodule(s) 3. Thyroid cyst 4. Thyroid cancer 5. Thyroid lymphoma	5.00 (0)	4.00‐5.00
14	1. Orbital cellulitis 2. Preseptal cellulitis (periorbital cellulitis) 3. Frontal sinusitis with subperiosteal abscess 4. Ethmoid sinusitis with orbital extension 5. Allergic rhinitis with a secondary bacterial infection	4.00 (1.00)	3.00‐5.00
15	1. Peritonsillar abscess 2. Infectious mononucleosis (Epstein‐Barr virus infection) 3. Bacterial tonsillitis 4. Lemierre's syndrome 5. Viral pharyngitis	5.00 (0)	4.00‐5.00
16	1. Vocal fold nodules 2. Muscle tension dysphonia 3. Chronic laryngitis 4. Reinke's edema (polypoid corditis) 5. Vocal fold paralysis or paresis	4.00 (1.00)	2.00‐5.00
17	1. Recurrent laryngeal nerve injury or palsy 2. Vocal cord paralysis 3. Aspiration pneumonia 4. Laryngeal edema 5. Laryngopharyngeal reflux	5.00 (1.00)	2.00‐5.00
18	1. Congenital muscular torticollis 2. Sternocleidomastoid tumor (fibromatosis colli) 3. Lymphatic malformation (cystic hygroma) 4. Branchial cleft cyst 5. Hemangioma	5.00 (1.00)	4.00‐5.00
19	1. Acute suppurative thyroiditis 2. Subacute (De Quervain's) thyroiditis 3. Lymphadenitis 4. Thyroglossal duct cyst infection 5. Abscess	3.00 (2.00)	1.00‐5.00
20	1. Acute otitis media with complications 2. Mastoiditis 3. Gradenigo's syndrome 4. Cholesteatoma with complications 5. Temporal bone osteomyelitis	4.00 (1.00)	2.00‐5.00
Overall		5.00 (1.00)	1.00‐5.00

Abbreviations: ChatGPT, chat generative pretrained transformer; IgG4, immunoglobulin G4; IQR, interquartile range.

Table 3 reports abbreviated management plans for the most likely differential diagnosis provided by ChatGPT, not the treatment plan best suited for the vignette. Full ChatGPT outputs are provided as supplemental information (Supplemental Figure S1, available online). Again, there was high agreement with the treatment plan for what ChatGPT felt was the most likely diagnosis with the vast majority achieving a median score of at least 4.00. The lowest median score was 3.00 for prompt #19. The highest median score was 5.00. The lowest IQR was 0 for prompts #6, #7, and #9. The highest IQR was 4.00 for prompt #5. Prompts #12 and #19 had an IQR of 3.00. Agreement with treatment was similarly high. Overall, the median agreement with treatment was 5.00 with an IQR of 1.00.

**Table 3:** ChatGPT‐Provided Treatment and Physician Agreement

Prompt	Treatment	Median (IQR)	Range
1	Based on the given scenario, the most likely diagnosis is an orbital blowout fracture. Here's a suggested treatment plan: *Consults*—ophthalmology, OMFS *Radiographic studies*—CT scan of orbits and facial bones *Medical treatment*—pain control, tetanus prophylaxis if immunization is not up to date, antibiotics may be prescribed *Surgical treatment*—depending on the extent of the fracture, muscle entrapment, and any functional or cosmetic concerns, surgical repair may be necessary within 1‐2 weeks of injury. Surgery typically involves reconstruction of the orbital floor or wall with implants or grafts, as well as the release of any entrapped muscles *Follow‐up*—regular follow‐up appointments with ophthalmology and maxillofacial surgery	4.00 (2.00)	1.00‐5.00
2	The most likely diagnosis, in this case, is acute bacterial sinusitis. Here's a suggested treatment plan: *Consults*—ENT if symptoms persist despite medical therapy *Radiographic studies*—CT sinus if there is no significant improvement within 48‐72 hours of starting antibiotics *Medical treatment*—antibiotics (amoxicillin‐clavulanate for 5‐10 days in adults; doxycycline or a respiratory fluoroquinolone if the patient has a penicillin allergy) *Symptomatic relief*—pain relief, oral or topical nasal decongestant no longer than 3 days to avoid rebound congestion, nasal saline irrigation *Follow up*—educate the patient on the importance of completing the full course of antibiotics, follow up in 10‐14 days	5.00 (1.00)	4.00‐5.00
3	Given the patient's history and presentation, the most likely diagnosis is squamous cell carcinoma (SCC) of the tongue. Here's a suggested treatment plan: *Consults*—ENT and OMFS *Radiographic studies*—contrast‐enhanced CT or MRI of the head and neck, CXR, or CT chest may be considered to rule out distant metastasis *Other studies*—biopsy of the lesion should be performed, along with imaging can be used to stage the tumor for treatment *Treatment*—may include a combination of surgery, radiation therapy, and chemotherapy	5.00 (1.00)	4.00‐5.00
	Surgery: resection with margins for early‐stage SCC of the tongue is the primary treatment; neck dissection if there is evidence of regional lymph node involvement or with advanced‐stage disease Radiation: postoperative radiation for high‐risk or advanced stage; definitive radiation as an alternative for patients who are not surgical candidates Chemotherapy: neoadjuvant or adjuvant chemotherapy for advanced stage or high risk of recurrence Follow up—regular follow‐up with ENT, OMFS, and oncologist		
4	The most likely diagnosis, in this case, is a spontaneous cerebrospinal fluid leak. Here's a suggested treatment plan: *Consults*—ENT and/or NSGY if CSF leak does not resolve with conservative measures *Radiographic studies*—high‐resolution CT of sinuses, MRI with intrathecal gadolinium *Other studies*—LP to measure opening pressure and obtain CSF for analysis *Medical treatment*—bed rest with head elevation, analgesics, and prophylactic antibiotics to minimize the risk of meningitis if necessary *Surgical treatment*—endoscopic surgical repair if the leak does not resolve with conservative measures, lumbar drain or shunt if the leak is not identifiable or if multiple leaks are present *Follow up*—weight loss/obesity management, monitor recurrence of symptoms which if persist or worsen then consider repeat imaging and re‐evaluating treatment plan	5.00 (2.00)	2.00‐5.00
5	The most likely diagnosis, in this case, is granulomatosis with polyangiitis. Here's a suggested treatment plan: *Consults*—rheumatology, ENT *Radiographic studies*— CXR, CT Chest *Labs*—CBC, CMP, ESR, CRP, ANCA, UA *Biopsy*—nasal or sinus biopsy *Medical treatment*—induction high‐dose corticosteroids with a DMARD; maintenance with less toxic DMARD *Follow up*—rheumatology, monitor laboratory markers, assess for end organ complications	4.00 (4.00)	1.00‐5.00
6	Based on the patient's history and presentation, the most likely diagnosis is a vestibular schwannoma (acoustic neuroma). Here's a suggested treatment plan: *Consults*—neuro‐otologist, NSGY, audiologist *Radiographic studies*—MRI head w/contrast *Treatment options*—observation (small tumors with mild symptoms), stereotactic radiosurgery (serviceable hearing and tumor < 3 cm), surgical resection (large tumors with significant symptoms or when hearing preservation is not possible) *Follow‐up*—facial nerve rehabilitation, neuro‐otology, NSGY	5.00 (0)	1.00‐5.00
7	The most likely diagnosis is Chronic Otitis Media with Cholesteatoma. Here is a treatment plan for this condition: *Consults*—ENT, audiologist *Radiographic studies*—high‐resolution CT of the temporal bones *Medical treatment*—treat concurrent infections with antibiotics and/or corticosteroid ear drops *Surgical treatment*—excision which may include canal‐wall‐up or canal‐wall‐down mastoidectomy with tympanoplasty *Follow up*—monitor for postoperative complications, regular follow up with otoscopic exams and audiograms, manage eustachian tube dysfunction and allergic rhinitis	5.00 (0)	1.00‐5.00
8	The most likely diagnosis, in this case, is pheochromocytoma with a cervical paraganglioma. Here's a treatment plan for this patient: *Consults*—endocrinology, ENT *Radiographic studies*—CT or MRI of the neck with contrast, abdominal CT or MRI *Labs*—plasma or 24‐hour urine metanephrines and catecholamines, CBC, renal function tests, and electrolytes *Medical treatment*—alpha‐blockers and subsequently beta‐blockers *Surgical treatment*—laparoscopic or open adrenalectomy and neck dissection *Follow‐up*—monitor patient's blood pressure and catecholeamine levels, adjust antihypertensive medications, imaging and laboratory testing for recurrence	4.00 (1.00)	2.00‐5.00
9	For the most likely diagnosis of auricular hematoma, the treatment plan would include the following steps: *Consults*—ENT *Treatment*—cold compress, aspiration or incision and drainage, pressure dressing or bolster after drainage, antibiotic prophylaxis, pain management *Follow‐up*—ENT within 1 week to monitor healing and removal of pressure dressing, avoid contact sports or other potentially traumatic activities until healing, and protective headgear in the future for prevention	5.00 (0)	4.00‐5.00
10	The most likely diagnosis, in this case, is sudden sensorineural hearing loss. Here's a treatment plan for this patient: *Consults*—ENT *Radiographic studies*—MRI brain and IAC with contrast *Labs*—CBC, renal function tests, electrolytes, autoimmune labs, and infectious etiologies (eg, Lyme disease, syphilitic) if indicated *Treatment*—oral corticosteroids preferably within 72 hours of onset with a 10‐14 day taper, intratympanic corticosteroid injections if oral treatment is contraindicated or ineffective, supportive care including hearing aids or assistive listening devices as necessary *Follow up*—follow up audiograms and adjust treatments as necessary	4.00 (1.00)	3.00‐5.00
11	The most likely diagnosis in this case is subglottic stenosis. The treatment plan for subglottic stenosis includes the following: *Consults*—ENT, pulmonology *Radiographic studies*—CT neck and chest *Other studies*—flexible laryngoscopy, pulmonary function tests *Medical treatment*—inhaled corticosteroids and bronchodilators *Surgical options*—endoscopic dilation, endoscopic lysis of adhesions, laser surgery, open surgical repair *Follow‐up*—speech therapy, pulmonary rehabilitation, regular follow‐up with ENT and pulmonology to monitor for recurrence	5.00 (1.00)	3.00‐5.00
12	The most likely diagnosis, in this case, is Sjögren's syndrome. Here's a treatment plan for this patient: *Consults*—rheumatology, ophthalmology *Radiographic studies*—CXR or CT test, parotid gland ultrasound *Labs*—CBC, renal function tests, electrolytes, liver function tests, ANA, anti‐Ro/SSA, anti‐La/SSB, ESR, CRP, immunoglobulins *Other studies*—Schirmer's test, minor salivary gland biopsy if clinically indicated *Treatment*—artificial tears and lubricating eye ointments, saliva substitutes, sugar‐free gum or lozenges, hydroxychloroquine, NSAIDs, pilocarpine, or cevimeline *Follow‐up*—close monitoring for potential complications, regular dental care, and good oral hygiene	4.00 (3.00)	1.00‐5.00
13	The most likely diagnosis, in this case, is a goiter associated with Hashimoto's thyroiditis. The treatment plan for goiter in the context of Hashimoto's thyroiditis includes the following: *Consults*—endocrinology *Radiographic studies*—thyroid ultrasound *Labs*—thyroid function tests *Other studies*—fine‐needle aspiration biopsy *Medical treatment*—levothyroxine for hypothyroidism and optionally for goiter size reduction *Surgical treatment*—thyroidectomy (partial or total) if there are compressive symptoms, malignancy is confirmed or strongly suspected, goiter is cosmetically bothersome *Follow‐up*—endocrinology, periodic thyroid ultrasound, and thyroid function tests	5.00 (1.00)	2.00‐5.00
14	The most likely diagnosis, in this case, is orbital cellulitis. Here's a treatment plan for this patient: *Consults*—ENT, ophthalmology *Radiographic studies*—CT or orbit and sinuses with contrast *Labs*—CBC, blood cultures, culture from sinus or conjunctival swab if possible *Medical treatment*—hospital admission, broad‐spectrum intravenous antibiotics to cover staphylococcus aureus and streptococcus species, adjust antibiotics when culture results are available, analgesics and antipyretics, nasal decongestants *Surgical treatment*—consider drainage of abscess or endoscopic sinus surgery in cases of severe infection, abscess, or lack of clinical improvement with conservative management *Follow‐up*—monitor for complications including vision loss, cavernous sinus thrombosis, and intracranial spread of infection	5.00 (1.00)	1.00‐5.00
15	The most likely diagnosis, in this case, is a peritonsillar abscess. Here is a suggested treatment plan: *Consults*—ENT *Radiographic studies*—CT neck with contrast *Labs*—CBC, blood cultures *Medical treatment*—intravenous hydration, intravenous broad‐spectrum antibiotics to cover group A *Streptococcus* and anaerobes, analgesics and antipyretics, adjust antibiotics based on culture and clinical response *Surgical treatment*—needle aspiration, incision, and drainage, or tonsillectomy *Follow‐up*—monitor patient's clinical response to treatment, follow up with ENT for recovery, and discuss elective tonsillectomy if recurrent tonsillitis is an issue	5.00 (1.00)	2.00‐5.00
16	The most likely diagnosis, in this case, is vocal fold nodules. Here's a treatment plan for this patient: *Consults*—ENT, speech‐language pathologist *Other studies*—laryngoscopy, videostroboscopy *Medical treatment*—voice therapy, vocal hygiene, voice rest, vocal warm‐ups and cool‐downs *Surgical treatment*—microlaryngoscopy with excision or laser ablation if conservative management fails to yield improvement or in severe cases *Follow‐up*—monitor for improvement with voice therapy and consider re‐evaluating diagnosis and exploring alternative treatments if there is no significant improvement in a few months	4.00 (1.00)	1.00‐5.00
17	Based on the most likely diagnosis of recurrent laryngeal nerve injury or palsy following coronary artery bypass graft surgery, I can suggest a general approach for management: *Consults*—ENT *Radiographic studies*—CT or MRI of neck and chest *Other studies*—laryngoscopy *Medical treatment*—voice and swallow therapy *Surgical treatment*—injection laryngoplasty, medialization thyroplasty, or arytenoid adduction *Follow‐up*—aspiration precautions, pneumonia prevention, follow‐up with ENT and speech‐language pathology	5.00 (1.00)	4.00‐5.00
18	The most likely diagnosis, in this case, is congenital muscular torticollis. Here's a treatment plan for this patient: *Consults*—pediatrician, physical therapist *Radiographic studies*—neck ultrasound *Medical treatment*—physical therapy, parent education for strengthening and stretching exercises, head positioning techniques *Surgical treatment*—surgical release of the sternocleidomastoid muscle in severe cases or cases where physical therapy fails to yield improvement *Follow‐up*—pediatrician, physical therapist	5.00 (1.00)	2.00‐5.00
19	Based on the most likely diagnosis of acute suppurative thyroiditis, I can suggest a general approach for management: *Consults*—endocrinology, ENT *Radiographic studies*—neck ultrasound, CT neck with contrast *Labs*—CBC, thyroid function tests *Other studies*—fine‐needle aspiration *Medical treatment*—intravenous broad‐spectrum antibiotics until culture results are available after which adjust the antibiotics for a 2‐3 week course, pain and fever management *Surgical treatment*—drainage of abscess or in rare cases thyroidectomy (partial or total) *Follow‐up*—endocrinology, ENT	3.00 (3.00)	1.00‐5.00
20	The most likely diagnosis, in this case, is acute otitis media with complications. Here's a treatment plan for this patient: *Consults*—ENT, audiology *Imaging studies*—CT of the temporal bone *Other studies*—CBC, CRP, ESR *Medical treatment*—broad‐spectrum antibiotics, analgesics, antipyretics *Surgical treatment*—myringotomy with or without tympanostomy tube placement or a mastoidectomy if complications such as mastoiditis or cholesteatoma are identified *Follow‐up*—close monitoring for improvement, complications, and need for surgery	4.00 (2.00)	2.00‐5.00
Overall		5.00 (1.00)	1.00‐5.00

Abbreviations: ANA, antinuclear antibody; ANCA, antineutrophil cytoplasmic antibody; CBC, complete blood count; ChatGPT, chat generative pretrained transformer; CMP, comprehensive metabolic panel; CRP, C‐reactive protein; CSF, cerebrospinal fluid; CT, computed tomography; CXR, chest X‐ray; DMARD, disease‐modifying antirheumatic drug; ENT, otolaryngology; ESR, erythrocyte sedimentation rate; IAC, internal auditory canal; IQR, interquartile range; LP, lumbar puncture; MRI, magnetic resonance imaging; NSAIDs, nonsteroidal anti‐inflammatory drugs; NSGY, neurosurgery; OMFS, oral maxillofacial surgery; SCC, squamous cell carcinoma; UA, urinanalysis.

To elucidate the potential limitations of ChatGPT in diagnosis and treatment, we performed univariate ordinal regression between prompt difficulty, diagnosis score, and treatment score. Strongly agree (Likert score of 5) was used as the reference level. Odds ratios with 95% confidence intervals and *p* value are presented in Table 4. The prompt difficulty was not a significant predictor of the diagnostic score as the odds of having a higher diagnostic score did not vary with increasing Likert score for prompt difficulty. Similarly, there was no association between prompt difficulty and treatment score. The odds of having a higher diagnostic score did not vary with an increasing Likert score for prompt difficulty. The diagnostic score did appear to be significantly related to the treatment score. Having a lower diagnostic score had lower odds of having a higher treatment score. More simply put, a lower diagnostic score was more likely associated with lower treatment scores. Odds ratios for diagnostic scores of 1, 2, 3, and 4 were 0.381, 0.330, 0.289, and 0.301 respectively. Diagnostic scores of 2 and 4 reach statistical significance (*p* < .001 and *p* = .050, respectively) while a score of 3 trended toward significance (*p* = .052). Having a Likert score of 1 likely did not reach significance (*p* = .307) due to the small sample size, as evidenced by the wide confidence interval for this level. Overall, this suggests that if there is no strong agreement with the diagnosis, agreement with treatment will also tend to be lower. Again, we emphasize that the treatment score is based on the top differential diagnosis selected by ChatGPT and not necessarily the diagnosis most otolaryngologists would select.

**Table 4:** Ordinal Regression Between Vignette Difficulty, Diagnostic Scores, and Treatment Scores

	Dependent: Diagnosis score Independent: Prompt difficulty			Dependent: Treatment score Independent: Prompt difficulty			Dependent: Treatment score Independent: Diagnosis score		
Likert score	OR	95% CI	*p* Value	OR	95% CI	*p* Value	OR	95% CI	*p* Value
1	2.01	(0.275, 14.30)	.495	0.482	(0.046, 5.00)	.542	0.381	(0.060, 2.44)	.307
2	0.853	(0.121, 5.99)	.874	0.273	(0.027, 2.80)	.274	0.330	(0.110, 1.00)	.050
3	0.865	(0.106, 7.03)	.892	0.868	(0.071, 10.70)	.912	0.289	(0.082, 1.01)	.052
4	0.660	(0.087, 5.00)	.162	0.232	(0.021, 2.53)	.231	0.301	(0.170, 0.533)	<.001
5	1	‐	‐	1	‐	‐	1	‐	‐

Abbreviations: CI, confidence interval; OR, odds ratio.

## Discussion

As a result of increasing data availability and accessibility to high‐performance AI technologies, AI is rapidly integrating across many industries, including health care. One of the most promising applications of AI in health care is diagnostics. The incorporation of publicly available and culturally ubiquitous AI systems, such as ChatGPT, will be inevitable in medicine. Whether the user is laymen or a clinician, it is imperative that physicians, particularly those in specialized care, understand its potential and limitations.

Several authors have begun to examine how ChatGPT might become involved in medicine. Some emphasize its role in supporting clinical tasks by helping create medical reports, patient‐specific forms and handouts, medical publishing, administration, and provider education.^6^ In fact, we used ChatGPT to assist in writing the introduction. While it required significant human editing, ChatGPT provided a strong framework to start with (Supplemental Figure S2, available online).

To date, no study has evaluated ChatGPT's performance in clinical otolaryngology, but several studies have found it to perform well in adjacent fields. Despite not being trained on a specific data set, ChatGPT performed at the level of a first‐year resident in plastic surgery on the in‐service training exam.^7^, ^8^ In neurosurgery, ChatGPT performed worse than the average user on Self‐Assessment Neurosurgery questions but better than residents in some topics.^9^ Clearly, there is already some rudimentary capacity in providing specialty care. This is consistent with the findings of our study which demonstrated a high rating from physicians for easy to moderately difficult clinical vignettes.

As evidenced in our study, when presented with clinical vignettes of well‐known clinical issues using medical jargon and curated relevant history, physical exam, and radiographic and laboratory findings, ChatGPT provides a very accurate differential diagnosis and reasonable treatment plans. This is likely due to the similarity of the vignettes provided in this study to the type of writing found in textbooks, scientific papers, and other data sources the AI model was trained on. This also explains why we failed to identify an association between prompt difficulty and diagnosis and treatment scores. ChatGPT performed better than the authors of this study expected for the difficulty of the prompts provided. If more difficult prompts were provided, we suggest that variations in diagnosis and treatment scores would become more apparent. Also, the apparent discordance between human‐assigned difficulty and the performance of the AI system is not surprising when we understand that the relative strengths and weaknesses between human intelligence and AI are different. Humans will tend to assign more difficulty to rare and esoteric conditions and treatments because the amount of data we can store is limited and we will prioritize more common and useful information. Computers do not have these limitations and can have access to more information than any person can have in their mind and obscurity is a trivial barrier for them. Nuanced diagnosis and treatment of more common clinical problems presented by patients in nonmedical jargon will likely be perceived as an easy problem by human evaluators but be difficult for AI systems.

Furthermore, it remains unclear how well it would perform if provided with real‐world, open‐ended free‐form histories, complete exams, and uncurated data which would often contain irrelevant, extraneous, and contradictory information. Unfiltered clinical information may prove too challenging for current LLMs to narrow into meaningful diagnoses, particularly in specialty fields. Several studies have demonstrated its impressive but inferior performance to human physicians in a variety of clinical vignettes.^5^, ^10^ Future studies may compare how ChatGPT performs with open‐ended inputs provided by otolaryngology patients (eg, “Why do I have nasal obstruction?”). Finally, some studies note that ChatGPT may generate different responses for the same prompt entered in multiplicate which undermines its clinical reliability.^11^

The role of ChatGPT from a patient's perspective in certain situations has been explored. ChatGPT is able to answer a variety of patient questions about colonoscopy in a digestible and generally satisfactory manner.^12^ It is also able to provide high‐quality answers to patient questions in pre‐ and postoperative care in oral and maxillofacial surgery, though the author suggests it be used in conjunction with surgeon experience.^13^ Within otolaryngology, ChatGPT provides patients with procedure‐specific instructions equivalent to institutional standards.^14^ However, ChatGPT instructions were equal to and inferior to Google's in terms of understandability and actionability, respectively.^14^ Patients are likely to find ChatGPT to be a useful and accessible resource. And while the quality of its answers for patients is surprisingly high, it cannot be used as a direct substitute for a physician's counseling. As alluded to above and by other authors, it may serve as a useful adjunct or starting point for patient handouts. Patients using ChatGPT should be counseled on these limitations. Furthermore, its use in guiding general practice providers in specialty care has not been assessed but remains an interesting concept.

Some limitations in ChatGPT may be inherent to its underlying data modeling. For instance, it lacks knowledge of events occurring after September 2021 and does not learn from its own experience.^2^ Additionally as its creators note ChatGPT may produce artificial hallucinations (ie, “produce content that is nonsensical or untruthful in relation to certain sources”) which can be difficult to discern when juxtaposed with ever‐increasing believability.^2^ Within biomedical research, this phenomenon is apparent when ChatGPT returns fake references when it is asked to cite its sources.^15^ How this impacts its capacity in medical decision‐making is not readily apparent.

Finally, from an ethics perspective, it should be reiterated that AI systems like ChatGPT in medicine are niche, and generalizability is still in its infancy. Moreover, unknown bias in ChatGPT can have significant and unintended consequences for patient outcomes. Inherent to AI is the training set they are based on which can in turn perpetuate disparities and biases in race, sex, and culture.^16^, ^17^, ^18^ Thus, the need for large amounts of high‐quality data that accurately and equitably represent a diverse patient population and understanding intrinsic bias when applying ChatGPT is imperative. Physicians that consider using ChatGPT in clinical practice must understand this aspect. And the burgeoning field of bioethics will also have to contemplate the risks and benefits of the use of AI in health care.^19^

## Conclusion

Overall, ChatGPT is a promising technology for both patients and physicians. Our study demonstrated that within otolaryngology, given highly curated vignettes, it provided differential diagnoses and treatment plans for easy to moderately difficult clinical scenarios that physicians highly agreed with. However, both physicians and patients need to be aware of the challenges and limitations of this LLM before implementing it in clinical practice. Physicians, medical societies, and patients, among other important stakeholders, should be involved in the development and application of these infant technologies in medicine.

## Author Contributions

**Roy W. Qu**, study design, data acquisition, statistical analysis, data interpretation, and manuscript preparation; **Uneeb Qureshi**, data acquisition, study design, data interpretation, and manuscript preparation; **Garrett Petersen**, data acquisition, study design, data interpretation, and manuscript preparation; **Steve C. Lee**, study design, data interpretation, and manuscript preparation. All authors approve the manuscript and agree to be accountable for all aspects of the work presented herein.

## Disclosures

### Competing interests

The authors have no conflicts of interest to declare.

### Funding source

None.

## Supporting information

Supporting information.

Supporting information.
