# Supplementary material for: Diagnostic and Management Applications of ChatGPT in Structured Otolaryngology Clinical Scenarios
Source: OTO Open. 2023 Aug 22;7(3):e67. doi: 10.1002/oto2.67 (PMC10442607; doi:10.1002/oto2.67)
Supplement: Supplementary file 1 — Supporting information. [file OTO2-7-e67-s001.docx]

**Supplemental Figure 1.**

ChatGPT full outputs as illustrated in the REDCap Survey and screenshot examples of inputs and outputs.


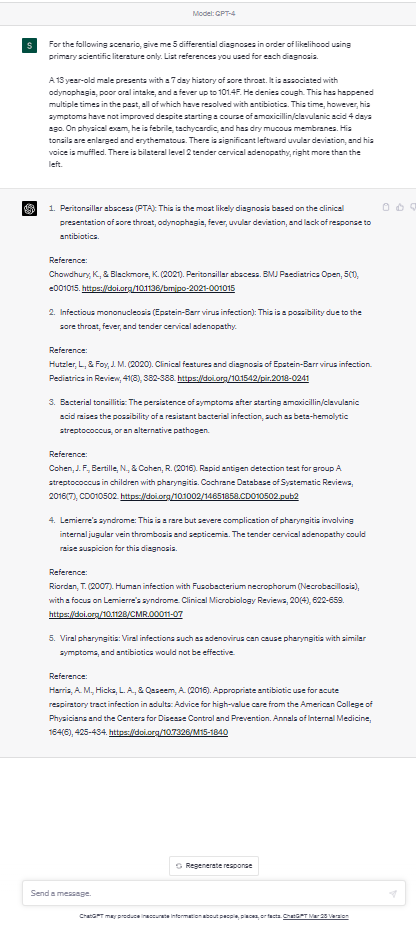


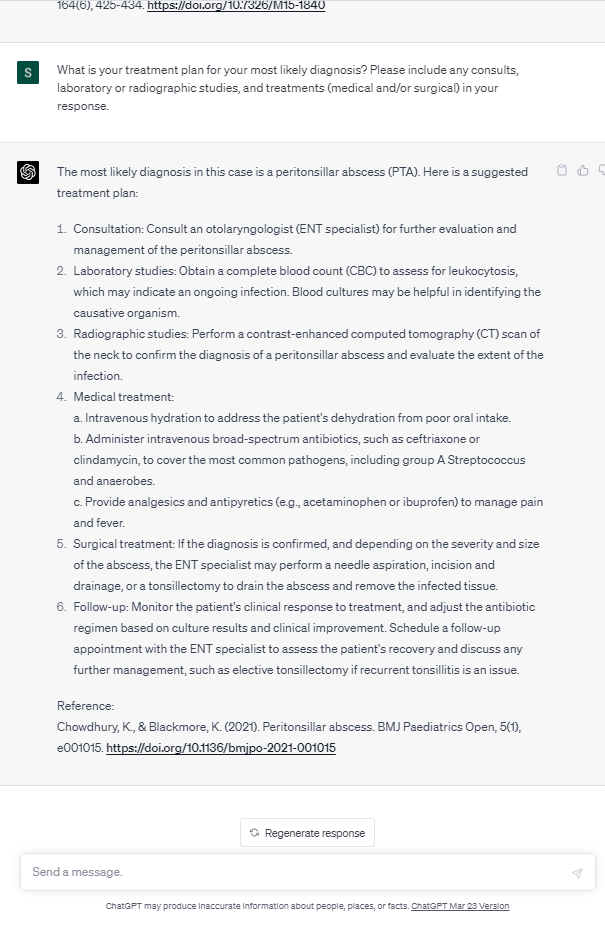


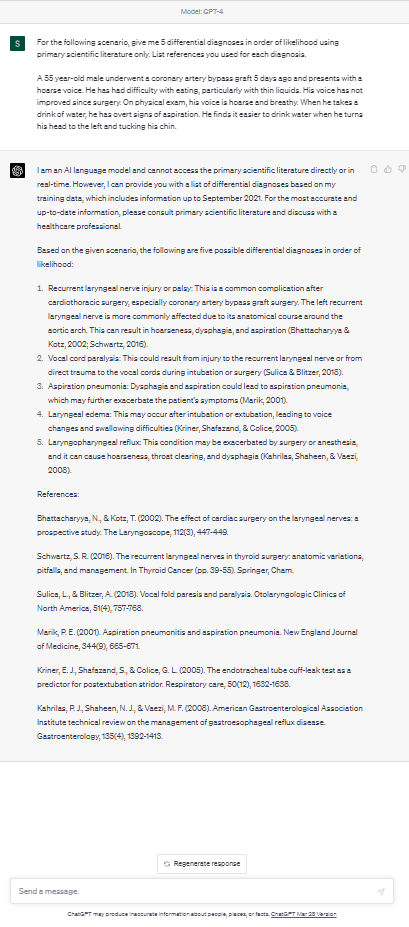

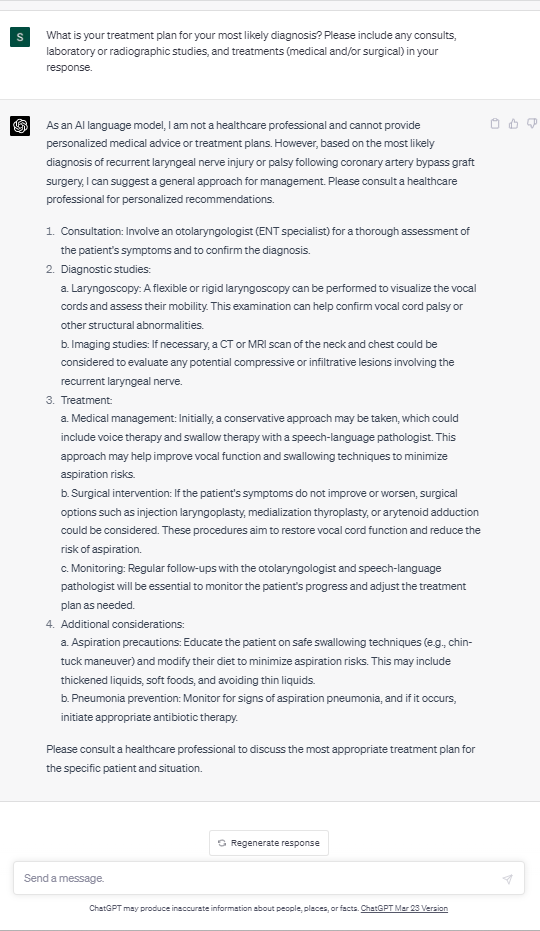


ChatGPT in Otolaryngology

**ChatGPT is an emerging, publicly available large language model artificial intelligence chatbot. It has raised significant interest in its application in medicine, including in diagnosis and treatment. We have fed ChatGPT 4.0 twenty (20) clinical prompts and asked it to generate the following information using primary scientific literature alone:**

1. **5 differential diagnoses**
2. **Treatment plan for the most likely diagnosis (including consults, laboratory or radiographic studies, and treatments (medical and/or surgical)**
3. Which choice best describes your subspecialty in Comprehensive Otolaryngology? Head & Neck

Facial Plastic and Reconstructive Neurotology


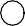

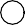

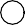

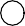

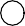

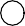

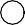


Pediatric Rhinology Laryngology

**Prompt #1:**

**A 25 year-old male was punched in his right eye while intoxicated at a bar. He reports right eye pain and double vision when looking up but denies vision loss. On physical exam, his right eye is soft and does not exhibit proptosis, but there is superior gaze restriction.**

1. How difficult would you rate this clinical prompt? Very easy Somewhat easy Neutral


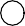

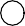

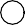

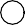

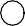


Somewhat difficult Very difficult

Differential Diagnoses:

- 1. Orbital blowout fracture
  2. Orbital contusion
  3. Superior rectus/levator palpebrae superioris muscle injury
  4. Cranial nerve palsy (specifically, oculomotor nerve palsy)
  5. Periorbital hematoma

1. How much do you agree with this list of differential Strongly disagree diagnoses? Somewhat disagree

Neutral Somewhat agree Strongly agree


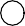

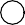

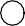

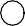

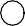


Treatment:

Based on the given scenario, the most likely diagnosis is an orbital blowout fracture. Here's a suggested treatment plan:

- 1. Consultation: An ophthalmologist and a maxillofacial surgeon should be consulted for a comprehensive evaluation and management of the injury.
  2. Radiographic studies: A CT scan of the orbits and facial bones should be performed to assess the extent of the fracture, involvement of adjacent structures, and any muscle entrapment.
  3. Medical treatment: Administer pain relief medications, such as acetaminophen or ibuprofen, for managing pain. Provide a tetanus prophylaxis if the patient's immunization is not up to date. Antibiotics may be prescribed to prevent infection, particularly if there are signs of an open fracture or involvement of the sinus.
  4. Surgical treatment: Depending on the extent of the fracture, muscle entrapment, and the presence of any functional or cosmetic concerns, surgical repair may be necessary. The timing of surgery is generally within 1-2 weeks of the injury to allow for the resolution of soft tissue swelling. Surgery typically involves the reconstruction of the orbital floor or wall with the use of implants or grafts, as well as the release of any entrapped muscles.
  5. Follow-up care: Regular follow-up appointments with the ophthalmologist and maxillofacial surgeon should be scheduled to monitor the healing process, evaluate eye movement, and address any complications.

1. For the diagnosis provided by ChatGPT, how much do you Strongly disagree agree with this treatment plan? Somewhat disagree

Neutral Somewhat agree Strongly agree


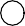

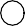

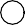

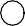

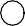


**Prompt #2:**

**A 21 year-old female presents with bilateral facial pain for 2 weeks. She states it started with a runny nose, cough and facial pressure 2 weeks ago. Her symptoms started to spontaneously improve after 7 days while taking Motrin. She started to develop worsening bilateral facial pain, particularly on her cheeks and forehead, associated with a fever and purulent nasal drainage. On physical exam, her frontal and maxillary sinuses are tender to palpation. There is green mucopurulent nasal drainage bilaterally, and there is edematous and erythematous mucosa throughout her nasal cavity.**

1. How difficult would you rate this clinical prompt? Very easy Somewhat easy Neutral


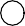

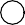

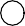

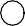

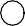


Somewhat difficult Very difficult

Differential Diagnoses:

- 1. Acute bacterial sinusitis
  2. Acute viral rhinosinusitis
  3. Allergic rhinitis
  4. Fungal sinusitis
  5. Dental infection with sinus involvement

1. How much do you agree with this list of differential Strongly disagree diagnoses? Somewhat disagree

Neutral Somewhat agree Strongly agree


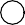

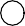

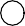

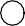

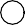


Treatment:

The most likely diagnosis in this case is acute bacterial sinusitis. Here's a suggested treatment plan:

- 1. Medical treatment:
     1. Antibiotics: Start the patient on empiric antibiotic therapy. Amoxicillin-clavulanate (500 mg/125 mg orally three times a day or 875 mg/125 mg orally twice a day) for 5-10 days is the first-line treatment in adults. If the patient is allergic to penicillin, consider doxycycline (100 mg orally twice a day) or a respiratory fluoroquinolone (e.g., levofloxacin 500 mg orally once daily) as an alternative.
  2. Symptomatic relief:
     1. Analgesics: Continue with ibuprofen (Motrin) as needed for pain relief.
     2. Decongestants: Recommend an oral or topical nasal decongestant, such as pseudoephedrine or oxymetazoline, to help alleviate nasal congestion. Advise the patient to use topical nasal decongestants for no more than 3 days to avoid rebound congestion.
     3. Nasal saline irrigation: Encourage the patient to perform nasal saline irrigation using a neti pot or saline spray to help clear nasal secretions and reduce inflammation.
  3. Additional studies and consults:
     1. If the patient does not show significant improvement within 48-72 hours of starting antibiotic therapy, consider ordering a computed tomography (CT) scan of the sinuses to evaluate for possible complications or alternative diagnoses.
     2. If the patient's symptoms persist despite appropriate medical treatment, consider referring the patient to an otolaryngologist (ENT specialist) for further evaluation and possible surgical intervention.
  4. Patient education and follow-up:
     1. Educate the patient on the importance of completing the full course of antibiotics, even if symptoms improve before the medication is finished.
     2. Schedule a follow-up appointment in 10-14 days to assess the patient's response to treatment and ensure resolution of symptoms.

1. For the diagnosis provided by ChatGPT, how much do you Strongly disagree agree with this treatment plan? Somewhat disagree


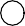

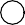

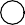

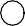

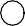


Neutral Somewhat agree Strongly agree

**Prompt #3:**

**A 65 year-old male with a 75 pack-year tobacco smoking history presents with a 5 cm, painful, ulcerative, and raised lateral tongue mass. He says it has continued to grow for the past 3 months and sometimes bleeds. On physical exam, the mass is friable, fixed and is isolated to the lateral ventral tongue. There are no palpable cervical lymph nodes.**

1. How difficult would you rate this clinical prompt? Very easy Somewhat easy Neutral


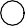

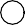

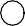

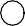

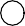


Somewhat difficult Very difficult

Differential Diagnoses:

- 1. Squamous cell carcinoma (SCC)
  2. Verrucous carcinoma: A rare, well-differentiated variant of SCC
  3. Oral pyogenic granuloma
  4. Major aphthous ulcer (Sutton's disease)
  5. Oral lichen planus

1. How much do you agree with this list of differential Strongly disagree diagnoses? Somewhat disagree

Neutral Somewhat agree Strongly agree


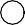

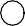

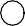

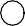

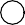


Treatment:

Given the patient's history and presentation, the most likely diagnosis is squamous cell carcinoma (SCC) of the tongue. Here's a suggested treatment plan:

- 1. Consultation: An otolaryngologist (ear, nose, and throat specialist) and an oral and maxillofacial surgeon should be consulted for a comprehensive evaluation and management of the lesion.
  2. Biopsy: A biopsy of the lesion should be performed to confirm the diagnosis of squamous cell carcinoma and to determine its grade and other histopathological features.
  3. Radiographic studies: Imaging studies, such as a contrast-enhanced CT scan or MRI of the head and neck, should be performed to evaluate the extent of the tumor, possible invasion of adjacent structures, and to assess for regional lymph node involvement. A chest X-ray or CT scan may be considered to rule out distant metastasis.
  4. Staging: Based on the results of the biopsy and imaging studies, the tumor should be staged according to the TNM classification system to guide treatment options.
  5. Treatment: The treatment plan for SCC of the tongue may include a combination of surgery, radiation therapy, and chemotherapy, depending on the stage of the disease and other factors.
     1. Surgery: Surgical resection of the tumor with adequate margins is the primary treatment for early-stage SCC of the tongue. Neck dissection may be performed if there is evidence of regional lymph node involvement or a high risk of occult metastasis.
     2. Radiation therapy: Postoperative radiation therapy may be indicated for high-risk patients or those with advanced-stage disease. In some cases, definitive radiation therapy may be an alternative to surgery for patients who are not surgical candidates or for those who refuse surgery.
     3. Chemotherapy: Neoadjuvant or adjuvant chemotherapy may be considered in patients with advanced-stage disease or those with a high risk of recurrence.
  6. Follow-up care: Regular follow-up appointments with the otolaryngologist, oral and maxillofacial surgeon, and oncologist should be scheduled to monitor the healing process, evaluate treatment response, and address any complications or recurrence.

1. For the diagnosis provided by ChatGPT, how much do you Strongly disagree agree with this treatment plan? Somewhat disagree


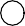

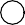

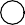

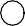

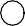


Neutral Somewhat agree Strongly agree

**Prompt #4:**

**A 33 year-old obese female with a 3 year history of bifrontal headache. These headaches are worse in the morning and when she lays down. She presents for evaluation of intermittent rhinorrhea for 3 months. It is clear, only comes from the left side, and does not have a particular taste. She has mild bilateral nasal congestion but denies facial pressure and change or loss of smell. She has tried intranasal steroid and anticholinergic nasal sprays which have not improved her symptoms. She was in a minor car accident 6 months ago, but the airbags were not deployed and she did not hit her head. On physical exam, there is no active nasal drainage in a head neutral or chin tucked position. Her nasal mucosa is not erythematous and there is no turbinate hypertrophy.**

1. How difficult would you rate this clinical prompt? Very easy Somewhat easy Neutral


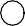

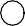

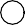

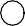

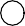


Somewhat difficult Very difficult

Differential Diagnoses:

- 1. Spontaneous cerebrospinal fluid (CSF) leak
  2. Idiopathic intracranial hypertension (pseudotumor cerebri)
  3. Chronic tension-type headache
  4. Migraine without aura
  5. Sinus headache (non-infectious)

1. How much do you agree with this list of differential Strongly disagree diagnoses? Somewhat disagree

Neutral Somewhat agree Strongly agree


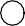

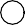

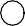

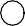

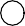


Treatment:

The most likely diagnosis in this case is spontaneous cerebrospinal fluid (CSF) leak. Here's a suggested treatment plan:

- 1. Diagnostic studies:
     1. High-resolution computed tomography (CT) scan of the sinuses to assess for bony defects and sinus abnormalities.
     2. Magnetic resonance imaging (MRI) with intrathecal gadolinium to identify the site of the CSF leak.
     3. Lumbar puncture to measure opening pressure and obtain CSF for analysis.
  2. Medical treatment:
     1. Bed rest with head elevation to reduce CSF pressure and promote spontaneous closure of the leak.
     2. Analgesics for headache management, such as acetaminophen or nonsteroidal anti-inflammatory drugs (NSAIDs).
     3. Prophylactic antibiotics to minimize the risk of meningitis, if necessary.
  3. Surgical intervention:
     1. If the leak does not resolve with conservative measures, consult an otolaryngologist (ENT specialist) for endoscopic surgical repair of the CSF leak.
     2. In cases where the site of the leak is not identifiable or if multiple leaks are present, a neurosurgeon may be consulted for the placement of a lumbar drain or shunt to divert CSF and alleviate pressure.
  4. Weight loss and lifestyle modifications:
     1. As obesity can be a risk factor for spontaneous CSF leaks, encourage the patient to adopt a healthy diet and exercise regimen to achieve a healthy weight.
     2. Provide education and resources on weight loss strategies, as well as the importance of maintaining a healthy weight to prevent future leaks.
  5. Follow-up:
     1. Schedule a follow-up appointment to assess the patient's response to treatment and monitor for any recurrence of symptoms.
     2. If symptoms persist or worsen, consider repeating imaging studies and reevaluating the treatment plan.

1. For the diagnosis provided by ChatGPT, how much do you Strongly disagree agree with this treatment plan? Somewhat disagree


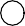

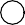

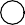

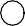

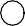


Neutral Somewhat agree Strongly agree

**Prompt #5:**

**A 22 year-old male presents with left-sided facial pain for 2 days. He has been feeling abnormally fatigued for the past 3 months, has had 20 lbs of unintended weight loss, and has been soaking through his sheets overnight. He has also noticed some bumps around his waist while putting on his belt. The facial pain is associated with mild chunky nasal drainage. He otherwise denies facial numbness and vision changes. On physical exam, he has a low grade fever of 99F. His facial sensation, extraocular movements, and visual acuity are intact. His conjunctiva is also clear. His left nasal cavity has some mild crusting and mucinous nasal drainage. The head of the left inferior turbinate head appears dusky, as does a 1 cm area of his left hard palate. He has reports normal sensation in these areas but these areas do not bleed when pricked. There is nontender inguinal lymphadenopathy.**

1. How difficult would you rate this clinical prompt? Very easy Somewhat easy Neutral


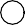

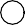

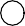

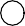

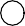


Somewhat difficult Very difficult

Differential Diagnoses:

- 1. Granulomatosis with polyangiitis (formerly known as Wegener's granulomatosis)
  2. Extranodal natural killer/T-cell lymphoma, nasal type
  3. Invasive fungal sinusitis (e.g., mucormycosis or aspergillosis)
  4. Tuberculosis (TB) infection with sinonasal involvement
  5. Syphilis with sinonasal manifestations

1. How much do you agree with this list of differential Strongly disagree diagnoses? Somewhat disagree

Neutral Somewhat agree Strongly agree


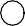

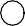

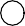

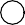

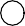


Treatment:

The most likely diagnosis in this case is granulomatosis with polyangiitis (GPA). Here's a suggested treatment plan:

- 1. Diagnostic studies:
     1. Complete blood count (CBC), comprehensive metabolic panel (CMP), and inflammatory markers (ESR, CRP).
     2. Anti-neutrophil cytoplasmic antibody (ANCA) testing, specifically PR3-ANCA.
     3. Chest X-ray or CT scan to evaluate for lung involvement.
     4. Nasal or sinus biopsy to confirm the presence of granulomatous inflammation and vasculitis.
     5. Urinalysis to evaluate for possible renal involvement.
  2. Consults:
     1. Rheumatologist for evaluation, confirmation of the diagnosis, and management of GPA.
     2. Otolaryngologist for further evaluation of nasal and sinus involvement, and possible surgical intervention if necessary.
  3. Medical treatment:
     1. Induction therapy: High-dose corticosteroids (e.g., prednisone 1 mg/kg/day) with a tapering schedule, combined with a disease-modifying anti-rheumatic drug (DMARD) such as rituximab or cyclophosphamide to induce remission.
     2. Maintenance therapy: After remission is achieved, continue with a less toxic DMARD, such as methotrexate, azathioprine, or mycophenolate mofetil, to maintain remission and prevent relapse.
  4. Monitoring and follow-up:
     1. Regular follow-up appointments with the rheumatologist to monitor disease activity, adjust medications, and manage side effects.
     2. Monitor laboratory markers (e.g., ANCA titers, inflammatory markers, renal function) to assess treatment response and detect potential relapses.
     3. Assess for possible complications or organ damage related to GPA and its treatment.

1. For the diagnosis provided by ChatGPT, how much do you Strongly disagree agree with this treatment plan? Somewhat disagree


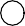

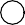

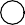

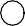

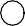


Neutral Somewhat agree Strongly agree

**Prompt #6:**

**A 40 year-old female comes in with a 12 month history of progressive right hearing loss. It is associated with a high frequency non-pulsatile tinnitus and intermittent vertigo. She denies otalgia and otorrhea. Recently, she has noticed the right-side of her face drooping over the past 3 months. On physical exam, her external auditory canals and tympanic membranes are clear. There is no middle ear effusion. Her tuning fork exam lateralizes to the left, and air conduction is greater than bone conduction bilaterally. She has a House-Brackmann grade 3 right facial paralysis.**

1. How difficult would you rate this clinical prompt? Very easy Somewhat easy Neutral


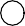

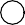

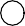

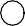

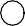


Somewhat difficult Very difficult

Differential Diagnoses:

- 1. Vestibular schwannoma (acoustic neuroma)
  2. Ramsay Hunt Syndrome (herpes zoster oticus)
  3. Ménière's disease
  4. Temporal bone fracture
  5. Autoimmune inner ear disease

1. How much do you agree with this list of differential Strongly disagree diagnoses? Somewhat disagree

Neutral Somewhat agree Strongly agree


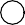

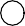

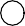

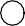

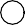


Treatment:

Based on the patient's history and presentation, the most likely diagnosis is a vestibular schwannoma (acoustic neuroma). Here's a suggested treatment plan:

- 1. Consultation: Consult with a neurotologist (ear and skull base specialist) and a neurosurgeon for a comprehensive evaluation and management of the lesion.
  2. Radiographic studies: Perform an MRI of the head with gadolinium contrast to visualize the lesion and assess its size, location, and involvement of adjacent structures, such as the facial and trigeminal nerves.
  3. Audiological evaluation: A comprehensive audiometric evaluation should be performed to assess the extent of hearing loss and to provide a baseline for comparison in the future.
  4. Treatment options: The management of vestibular schwannomas depends on the size of the tumor, the degree of hearing loss, the severity of symptoms, and the patient's overall health. Treatment options include observation, stereotactic radiosurgery, and surgical resection.
     1. Observation: Small tumors with mild symptoms and stable hearing may be managed conservatively with periodic MRI and audiological monitoring.
     2. Stereotactic radiosurgery: This is a non-invasive treatment option for small to medium-sized tumors that uses precisely targeted radiation to control tumor growth. It may be considered for patients with serviceable hearing and tumors less than 3 cm in diameter.
     3. Surgical resection: Surgical removal of the tumor may be considered for larger tumors, those causing significant symptoms, or in cases where hearing preservation is not possible. The choice of surgical approach (e.g., translabyrinthine, retrosigmoid, or middle fossa) depends on tumor size, hearing status, and surgeon preference.
  5. Facial nerve rehabilitation: For patients with facial paralysis, referral to a facial nerve specialist and physical therapy may be beneficial for facial reanimation and rehabilitation.
  6. Follow-up care: Regular follow-up appointments with the neurotologist and neurosurgeon should be scheduled to monitor treatment response, evaluate for tumor recurrence, and address any complications

1. For the diagnosis provided by ChatGPT, how much do you Strongly disagree agree with this treatment plan? Somewhat disagree


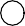

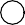

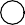

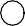


Neutral Somewhat agree Strongly agree

**Prompt #7:**

**A 34 year-old male with a history of allergic rhinitis, eustachian tube dysfunction, and recurrent episodes of right acute otitis media presents with 6 months of progressive right sided hearing loss and clear otorrhea. He denies otalgia, vertigo, and tinnitus. On physical exam, the right external auditory canal is clear. The right tympanic membrane has a superior retraction pocket with a possible tympanic membrane perforation, erosion of the malleus, and a small serous middle ear effusion. There is also a round, pearlescent mass in the right middle ear. His left external auditory canal and tympanic membrane are clear. His tuning fork exam lateralizes to the right, and air conduction is greater than bone conduction on the left but bone conduction is greater than air conduction on the right. His facial nerve is intact.**

1. How difficult would you rate this clinical prompt? Very easy Somewhat easy Neutral

Somewhat difficult Very difficult

Differential Diagnoses:

- 1. Chronic Otitis Media (COM) with Cholesteatoma
  2. Chronic Serous Otitis Media
  3. Adhesive Otitis Media
  4. Tympanosclerosis
  5. Congenital Cholesteatoma

1. How much do you agree with this list of differential Strongly disagree diagnoses? Somewhat disagree

Neutral Somewhat agree Strongly agree

Treatment:

The most likely diagnosis is Chronic Otitis Media (COM) with Cholesteatoma. Here is a treatment plan for this condition:

- 1. Consultation: Refer the patient to an otolaryngologist (ear, nose, and throat specialist) for a comprehensive evaluation and management of the cholesteatoma.
  2. Radiographic Studies: Order a high-resolution computed tomography (CT) scan of the temporal bones to assess the extent of the cholesteatoma, any bone erosion, and possible complications.
  3. Audiometry: Perform a comprehensive audiogram to determine the extent of hearing loss in both ears and to establish a baseline for future evaluations.
  4. Medical Management: Treat any concurrent infections or inflammation with appropriate antibiotics and/or corticosteroid ear drops as directed by the otolaryngologist.
  5. Surgical Management: The definitive treatment for cholesteatoma is surgical removal. The specific surgical approach will depend on the size, location, and extent of the cholesteatoma, as well as the patient's hearing status. Common surgical procedures include canal-wall-up (CWU) or canal-wall-down (CWD) mastoidectomy with tympanoplasty. The primary goal is to remove the cholesteatoma and prevent recurrence, while preserving or improving the patient's hearing.
  6. Postoperative Care: Monitor the patient postoperatively for any complications such as infection, hearing loss, or facial nerve dysfunction. Perform regular follow-up appointments, including otoscopic examinations and audiograms, to ensure the cholesteatoma has not recurred and to monitor the patient's hearing status.
  7. Long-term Management: Educate the patient on the importance of regular follow-up appointments with the otolaryngologist to monitor for any recurrence of cholesteatoma, as well as the proper management of eustachian tube dysfunction and any underlying conditions, such as allergic rhinitis, to minimize the risk of future ear infections and complications.

1. For the diagnosis provided by ChatGPT, how much do you Strongly disagree agree with this treatment plan? Somewhat disagree

Neutral Somewhat agree Strongly agree

**Prompt #8:**

**A 34 year-old male with no past medical history presents with left-sided neck mass for the past year. It has been slowly growing for the past year and is nontender. During the same time, he has noticed episodes of spontaneous palpitations and sweating. During a visit with his primary care doctor last month, his blood pressure was noted to be 190/110s, and he was sent to the emergency room. On physical exam, there is a firm 3cm mobile left level 2 neck mass. There are no other palpable masses, including thyroid masses. His oral cavity and oropharynx exam are normal and his vital signs are normal.**

1. How difficult would you rate this clinical prompt? Very easy Somewhat easy Neutral

Somewhat difficult Very difficult

Differential Diagnoses:

- 1. Pheochromocytoma with a cervical paraganglioma
  2. Cervical lymphadenopathy due to metastatic malignancy
  3. Schwannoma of the cervical sympathetic chain
  4. Branchial cleft cyst
  5. Tuberculous lymphadenitis

1. How much do you agree with this list of differential Strongly disagree diagnoses? Somewhat disagree

Neutral Somewhat agree Strongly agree

Treatment:

The most likely diagnosis in this case is pheochromocytoma with a cervical paraganglioma. Here's a treatment plan for this patient:

- 1. Consults:
     1. Endocrinology: to evaluate, confirm the diagnosis, and manage the hypertension related to the pheochromocytoma.
     2. Otolaryngology/Head and Neck Surgery: for assessment and potential surgical management of the cervical paraganglioma.
  2. Laboratory studies:
     1. Plasma or 24-hour urinary metanephrines and catecholamines: to confirm the presence of a pheochromocytoma.
     2. Complete blood count, renal function tests, and electrolytes: to assess the patient's general health and readiness for potential surgery.
  3. Radiographic studies:
     1. Contrast-enhanced computed tomography (CT) scan or magnetic resonance imaging (MRI) of the neck: to evaluate the extent and location of the cervical paraganglioma.
     2. Abdominal CT scan or MRI: to identify and locate the pheochromocytoma.
  4. Treatment:
     1. Medical management: Alpha-blockers (e.g., phenoxybenzamine) should be initiated to control hypertension and prevent hypertensive crisis during the perioperative period. Beta-blockers may be added subsequently if needed to manage tachycardia.
     2. Surgical management: Once the patient is medically stable and prepared for surgery, laparoscopic or open adrenalectomy can be considered for pheochromocytoma removal, and a neck dissection can be performed to excise the cervical paraganglioma. The surgery should be performed by experienced surgeons due to the risk of intraoperative complications related to catecholamine release.
     3. Postoperative management: Monitor the patient's blood pressure and catecholamine levels, and adjust antihypertensive medications as needed. Provide appropriate follow-up care, including imaging and laboratory testing, to ensure no recurrence of the paraganglioma or pheochromocytoma.

1. For the diagnosis provided by ChatGPT, how much do you Strongly disagree agree with this treatment plan? Somewhat disagree

Neutral Somewhat agree Strongly agree

**Prompt #9:**

**A 25 year-old female presents with left otalgia 4 hours after a boxing match where she sustained a strike to the left side of her head. She states it has become swollen and increasingly swollen. On physical exam, the helix and antihelix of the left pinna are swollen, fluctuant and have mild ecchymosis. The external auditory canal and tympanic membrane are clear bilaterally.**

1. How difficult would you rate this clinical prompt? Very easy Somewhat easy Neutral

Somewhat difficult Very difficult

Differential Diagnoses:

- 1. Auricular hematoma
  2. Perichondritis
  3. Traumatic ear laceration
  4. Acute otitis externa
  5. Temporomandibular joint (TMJ) dysfunction

1. How much do you agree with this list of differential Strongly disagree diagnoses? Somewhat disagree

Neutral Somewhat agree Strongly agree

Treatment:

For the most likely diagnosis of auricular hematoma, the treatment plan would include the following steps:

- 1. Immediate treatment:
- Apply a cold compress to the affected area to reduce swelling and alleviate pain.
  1. Consultation:
- Consult an otolaryngologist (ENT specialist) for further evaluation and management.
  1. Aspiration or incision and drainage:
- The otolaryngologist may perform an aspiration or incision and drainage of the hematoma to prevent complications such as cartilage necrosis, infection, or cauliflower ear deformity. Local anesthesia is typically administered before the procedure.
  1. Pressure dressing:
- After the hematoma has been drained, a pressure dressing or bolster may be applied to the ear to prevent reaccumulation of blood and to promote healing.
  1. Antibiotic prophylaxis:
- Since the risk of infection is increased after incision and drainage, an oral antibiotic prophylaxis, such as cephalexin or amoxicillin-clavulanate, may be prescribed to prevent infection.
  1. Pain management:
- Prescribe oral analgesics such as acetaminophen or ibuprofen to manage pain.
  1. Follow-up care:
- Schedule a follow-up appointment with the otolaryngologist within a week to monitor healing and remove the pressure dressing.
  1. Prevention:
- Advise the patient to avoid contact sports or other activities that may cause trauma to the ear until the area has healed completely. In the future, wearing protective headgear during contact sports like boxing may help prevent recurrence of auricular hematoma.

1. For the diagnosis provided by ChatGPT, how much do you Strongly disagree agree with this treatment plan? Somewhat disagree

Neutral Somewhat agree Strongly agree

**Prompt #10:**

**A 29 year-old male presents with 2 day history of right hearing loss. He was working at a coffee shop and noticed right-sided hearing loss when he went home 2 days ago. He was not doing anything out of the ordinary that day. He recently came back from a trip from Colorado 1 week ago where he spent most of his time skiing. He has no other complaints and has not had hearing problems prior to 2 days ago. On physical exam, his pinna, external auditory canal, and tympanic membrane are normal. He brings an audiogram obtained yesterday that shows a 30 dB hearing loss from 250-3000Hz and a downsloping 30-60dB hearing loss from 4000-8000Hz in the right ear. Air and bone conduction thresholds are equivalent. Hearing in the left ear is normal.**

1. How difficult would you rate this clinical prompt? Very easy Somewhat easy Neutral

Somewhat difficult Very difficult

Differential Diagnoses:

- 1. Sudden sensorineural hearing loss (SSNHL)
  2. Acoustic trauma
  3. Barotrauma
  4. Eustachian tube dysfunction
  5. Meniere's disease (early stage)

1. How much do you agree with this list of differential Strongly disagree diagnoses? Somewhat disagree

Neutral Somewhat agree Strongly agree

Treatment:

The most likely diagnosis in this case is sudden sensorineural hearing loss (SSNHL). Here's a treatment plan for this patient:

- 1. Consults:
     1. Otolaryngology: for evaluation and management of SSNHL.
  2. Laboratory studies:
     1. Consider ordering a complete blood count, renal function tests, and electrolytes to assess the patient's general health.
     2. Evaluate for autoimmune causes and infectious etiologies (e.g., Lyme disease, syphilis) if clinically indicated.
  3. Radiographic studies:
     1. Magnetic resonance imaging (MRI) of the brain and internal auditory canals with gadolinium contrast: to rule out retrocochlear pathology, such as vestibular schwannoma or other central lesions.
  4. Treatment:
     1. Medical management: Initiate oral corticosteroids, such as prednisone, as soon as possible (preferably within 72 hours of symptom onset). Taper the corticosteroids over a 10-14 day period. Monitor for potential side effects and contraindications to corticosteroid use.
     2. If oral corticosteroids are contraindicated or ineffective, consider intratympanic corticosteroid injections as an alternative treatment option.
     3. Provide supportive care, including hearing aids or assistive listening devices, as needed.
     4. Monitor the patient's progress with follow-up audiograms and adjust treatment as necessary.

1. For the diagnosis provided by ChatGPT, how much do you Strongly disagree agree with this treatment plan? Somewhat disagree

Neutral Somewhat agree Strongly agree

**Prompt #11:**

**A 37 year-old female presents with progressive shortness of breath. 5 months ago, she was in a housefire where she sustained severe inhalational injuries and was intubated for 14 days.**

**After that incident, she has had progressive shortness of breath and has recently developed noisy, high-pitched breathing over the past 3 weeks that is worse when she exercises. On physical exam, her voice is hoarse and weak. She has mild suprasternal retractions, biphasic stridor, and requires 2 liters/min of supplemental oxygen to maintain oxygen saturations**

**above 92%.**

1. How difficult would you rate this clinical prompt? Very easy Somewhat easy Neutral

Somewhat difficult Very difficult

Differential Diagnoses:

- 1. Subglottic stenosis
  2. Tracheomalacia
  3. Vocal cord dysfunction
  4. Bronchiolitis obliterans
  5. Reactive airway dysfunction syndrome (RADS)

1. How much do you agree with this list of differential Strongly disagree diagnoses? Somewhat disagree

Neutral Somewhat agree Strongly agree

Treatment:

The most likely diagnosis in this case is subglottic stenosis. The treatment plan for subglottic stenosis includes the following:

- 1. Consultations:
- Refer the patient to an otolaryngologist (ENT) for a thorough evaluation and management of her airway.
- Involve a pulmonologist to optimize her respiratory function and address any coexisting pulmonary issues.
  1. Diagnostic studies:
- Flexible laryngoscopy: To visualize the vocal cords and the subglottic area, assessing the severity and location of the stenosis.
- CT scan of the neck and chest: To evaluate the extent of stenosis and surrounding structures.
- Pulmonary function tests: To assess the patient's lung function and identify any coexisting pulmonary disorders.
  1. Medical treatment:
- Inhaled corticosteroids and bronchodilators: To decrease inflammation and improve airflow if there is any coexisting reactive airway disease or asthma.
  1. Surgical intervention: The choice of surgical intervention depends on the severity and location of the stenosis.
- Endoscopic dilation: This procedure involves using a balloon or bougie dilator to expand the narrowed area in the airway. It may require multiple sessions for optimal results.
- Endoscopic lysis of adhesions and scar tissue: This can help improve the airway opening in some cases.
- Laser surgery: CO2 laser or other laser types can be used to ablate scar tissue and widen the airway.
- Open surgical repair: In severe cases, a laryngotracheal reconstruction or cricotracheal resection may be necessary to reestablish a patent airway.
  1. Postoperative care:
- Speech therapy: To address the hoarseness and improve vocal cord function.
- Pulmonary rehabilitation: To optimize the patient's respiratory function and help her regain her exercise capacity.
- Close follow-up: Regular follow-up with the otolaryngologist and pulmonologist to monitor her airway and respiratory status, and to address any complications or recurrence.

1. For the diagnosis provided by ChatGPT, how much do you Strongly disagree agree with this treatment plan? Somewhat disagree

Neutral Somewhat agree Strongly agree

**Prompt #12:**

**A 39 year-old female presents with a history of recurrent bilateral cheek swelling. These episodes are accompanied by intermittent low grade fever, up to 100F. She has had these symptoms for the past 2 months. She has also had to drink more water during these episodes, particularly when she eats. She is currently undergoing a work up for shortness of breath and pulmonary hilar fullness discovered on a chest X-ray 3 months ago. Sometimes she has vision changes and facial weakness with these episodes, but these are rare. She states she is currently feeling normal. On physical, she is afebrile. Her parotid and submandibular glands are normal to palpable, and there is no palpable cervical lymphadenopathy.**

1. How difficult would you rate this clinical prompt? Very easy Somewhat easy Neutral

Somewhat difficult Very difficult

Differential Diagnoses:

- 1. Sjögren's syndrome
  2. Sarcoidosis
  3. Mikulicz's disease (IgG4-related disease)
  4. Recurrent parotitis
  5. Lymphoma

1. How much do you agree with this list of differential Strongly disagree diagnoses? Somewhat disagree

Neutral Somewhat agree Strongly agree

Treatment:

The most likely diagnosis in this case is Sjögren's syndrome. Here's a treatment plan for this patient:

- 1. Consults:
     1. Rheumatology: for evaluation, confirmation of the diagnosis, and management of Sjögren's syndrome.
     2. Ophthalmology: for assessment and management of potential ocular complications related to Sjögren's syndrome.
  2. Laboratory studies:
     1. Complete blood count, renal function tests, electrolytes, and liver function tests: to assess the patient's general health.
     2. Antinuclear antibodies (ANA), anti-Ro/SSA, and anti-La/SSB: to support the diagnosis of Sjögren's syndrome.
     3. Erythrocyte sedimentation rate (ESR) and C-reactive protein (CRP): to evaluate for systemic inflammation.
     4. Immunoglobulins: to assess for potential immunodeficiency.
  3. Radiographic studies:
     1. Chest X-ray or computed tomography (CT) scan: to further evaluate hilar fullness and potential pulmonary involvement.
     2. Parotid gland ultrasonography: to assess for glandular abnormalities.
  4. Additional diagnostic studies:
     1. Schirmer's test and tear break-up time: to evaluate tear production and ocular dryness.
     2. Minor salivary gland biopsy: to confirm the diagnosis of Sjögren's syndrome, if clinically indicated.
  5. Treatment:
     1. Symptomatic management: Artificial tears and lubricating ointments for dry eyes; saliva substitutes, sugar-free gum, or lozenges for dry mouth.
     2. Hydroxychloroquine: for fatigue, arthralgia, and systemic symptoms associated with Sjögren's syndrome.
     3. Nonsteroidal anti-inflammatory drugs (NSAIDs): for pain and inflammation management.
     4. Pilocarpine or cevimeline: to stimulate saliva production.
     5. Close monitoring for potential complications, including infections, and organ involvement.
     6. Regular dental care and good oral hygiene to prevent dental caries and infections.

1. For the diagnosis provided by ChatGPT, how much do you Strongly disagree agree with this treatment plan? Somewhat disagree

Neutral Somewhat agree Strongly agree

**Prompt #13:**

**A 60 year-old female with a history of Hashimoto's thyroiditis presents with a 1 year history of an enlarging neck. She has noticed the left side of her neck become more prominent, which bothers her, and has found it more difficult to swallow solids in the past 3 months. She has not had unintended weight loss. On physical exam, she has a 6 cm nodular neck mass, just left of midline, that moves with swallowing. There is no palpable lymphadenopathy, and her voice is normal.**

1. How difficult would you rate this clinical prompt? Very easy Somewhat easy Neutral

Somewhat difficult Very difficult

Differential Diagnoses:

- 1. Goiter associated with Hashimoto's thyroiditis
  2. Thyroid nodule(s)
  3. Thyroid cyst
  4. Thyroid cancer
  5. Thyroid lymphoma

1. How much do you agree with this list of differential Strongly disagree diagnoses? Somewhat disagree

Neutral Somewhat agree Strongly agree

Treatment:

The most likely diagnosis in this case is a goiter associated with Hashimoto's thyroiditis. The treatment plan for a goiter in the context of Hashimoto's thyroiditis includes the following:

- 1. Consultations:
- Refer the patient to an endocrinologist for a comprehensive evaluation and management of her thyroid function and goiter.
  1. Diagnostic studies:
- Thyroid function tests (TFTs): Assess the patient's thyroid hormone levels (TSH, free T4, and free T3) to determine if she has hypothyroidism, euthyroidism, or hyperthyroidism.
- Thyroid ultrasound: Evaluate the size, structure, and characteristics of the thyroid gland, the goiter, and any nodules.
- Fine-needle aspiration biopsy (FNAB): If there are suspicious nodules or features on ultrasound, an FNAB should be performed to rule out malignancy.
  1. Medical treatment:
- Levothyroxine: If the patient is found to have hypothyroidism, initiate treatment with levothyroxine to normalize her TSH levels, which may help reduce the goiter size.
- If the patient is euthyroid or has subclinical hypothyroidism, the use of levothyroxine may still be considered to potentially reduce the size of the goiter, but should be individualized based on the patient's specific case.
  1. Surgical intervention: Surgery may be indicated in certain situations, such as:
- If the goiter is causing compressive symptoms (e.g., dysphagia, breathing difficulties).
- If malignancy is confirmed or strongly suspected.
- If the goiter is cosmetically bothersome to the patient and medical management has not led to a significant reduction in size.
- In such cases, consult with a thyroid surgeon to discuss the appropriateness of a thyroidectomy (partial or total) for the patient.
  1. Monitoring and follow-up:
- Regular follow-up with the endocrinologist is important to monitor the patient's thyroid function, goiter size, and any potential complications.
- Periodic thyroid ultrasounds and thyroid function tests should be performed to assess for changes in the goiter or the patient's thyroid status.

1. For the diagnosis provided by ChatGPT, how much do you Strongly disagree agree with this treatment plan? Somewhat disagree

Neutral Somewhat agree Strongly agree

**Prompt #14:**

**A 4 year-old male presents with double vision for 1 day. His older brother developed a cough and nasal congestion 2 weeks ago which improved without intervention after 5 days. One week ago, the patient developed similar symptoms and nasal congestion. He woke up with a headache and forehead swelling this morning. On physical exam, he is febrile to 101.2F. His forehead is tense, swollen, tender and mildly fluctuant. His right eye has mild conjunctival injection, has mild proptosis. There is mild right-sided abduction with lateral gaze, but visual acuity is intact. There is mild clear bilateral mucinous rhinorrhea. There is no neck stiffness.**

1. How difficult would you rate this clinical prompt? Very easy Somewhat easy Neutral

Somewhat difficult Very difficult

Differential Diagnoses:

- 1. Orbital cellulitis
  2. Preseptal cellulitis (Periorbital cellulitis)
  3. Frontal sinusitis with subperiosteal abscess
  4. Ethmoid sinusitis with orbital extension
  5. Allergic rhinitis with secondary bacterial infection

1. How much do you agree with this list of differential Strongly disagree diagnoses? Somewhat disagree

Neutral Somewhat agree Strongly agree

Treatment:

The most likely diagnosis in this case is orbital cellulitis. Here's a treatment plan for this patient:

- 1. Consults:
     1. Ophthalmology: for evaluation and management of orbital cellulitis.
     2. Otolaryngology: for assessment and management of potential sinus infection.
  2. Laboratory studies:
     1. Complete blood count: to assess for systemic infection and inflammation.
     2. Blood cultures: to identify potential causative organisms.
     3. If possible, obtain a culture from the affected area (e.g., sinus or conjunctival swab).
  3. Radiographic studies:
     1. Contrast-enhanced computed tomography (CT) of the orbits and sinuses: to assess the extent of infection, rule out complications, and identify any associated sinusitis.
  4. Treatment:
     1. Hospital admission: for close monitoring and management of orbital cellulitis.
     2. Intravenous broad-spectrum antibiotics: covering common pathogens like Staphylococcus aureus and Streptococcus species. Adjust antibiotics based on culture results and sensitivities when available.
     3. Analgesics and antipyretics: to manage fever and pain.
     4. Nasal decongestants: to relieve nasal congestion and promote sinus drainage.
     5. Close monitoring for potential complications, including vision loss, cavernous sinus thrombosis, and intracranial spread of infection.
  5. Surgical intervention:
     1. Consider surgical intervention (e.g., drainage of abscess or endoscopic sinus surgery) in cases of severe infection, abscess formation, or lack of clinical improvement with conservative management.

1. For the diagnosis provided by ChatGPT, how much do you Strongly disagree agree with this treatment plan? Somewhat disagree

Neutral Somewhat agree Strongly agree

**Prompt #15:**

**A 13 year-old male presents with a 7 day history of sore throat. It is associated with odynophagia, poor oral intake, and a fever up to 101.4F. He denies cough. This has happened multiple times in the past, all of which have resolved with antibiotics. This time, however, his symptoms have not improved despite starting a course of amoxicillin/clavulanic acid 4 days ago. On physical exam, he is febrile, tachycardic, and has dry mucous membranes. His tonsils are enlarged and erythematous. There is significant leftward uvular deviation, and his voice is muffled. There is bilateral level 2 tender cervical adenopathy, right more than the left.**

1. How difficult would you rate this clinical prompt? Very easy Somewhat easy Neutral

Somewhat difficult Very difficult

Differential Diagnoses:

- 1. Peritonsillar abscess (PTA)
  2. Infectious mononucleosis (Epstein-Barr virus infection)
  3. Bacterial tonsillitis
  4. Lemierre's syndrome
  5. Viral pharyngitis

1. How much do you agree with this list of differential Strongly disagree diagnoses? Somewhat disagree

Neutral Somewhat agree Strongly agree

Treatment:

The most likely diagnosis in this case is a peritonsillar abscess (PTA). Here is a suggested treatment plan:

- 1. Consultation: Consult an otolaryngologist (ENT specialist) for further evaluation and management of the peritonsillar abscess.
  2. Laboratory studies: Obtain a complete blood count (CBC) to assess for leukocytosis, which may indicate an ongoing infection. Blood cultures may be helpful in identifying the causative organism.
  3. Radiographic studies: Perform a contrast-enhanced computed tomography (CT) scan of the neck to confirm the diagnosis of a peritonsillar abscess and evaluate the extent of the infection.
  4. Medical treatment: a. Intravenous hydration to address the patient's dehydration from poor oral intake. b. Administer intravenous broad-spectrum antibiotics, such as ceftriaxone or clindamycin, to cover the most common pathogens, including group A Streptococcus and anaerobes. c. Provide analgesics and antipyretics (e.g., acetaminophen or ibuprofen) to manage pain and fever.
  5. Surgical treatment: If the diagnosis is confirmed, and depending on the severity and size of the abscess, the ENT specialist may perform a needle aspiration, incision and drainage, or a tonsillectomy to drain the abscess and remove the infected tissue.
  6. Follow-up: Monitor the patient's clinical response to treatment, and adjust the antibiotic regimen based on culture results and clinical improvement. Schedule a follow-up appointment with the ENT specialist to assess the patient's recovery and discuss any further management, such as elective tonsillectomy if recurrent tonsillitis is an issue.

1. For the diagnosis provided by ChatGPT, how much do you Strongly disagree agree with this treatment plan? Somewhat disagree

Neutral Somewhat agree Strongly agree

**Prompt #16:**

**A 42 year old female presents with a 1 year history of a hoarse and strained voice. She reports a 2 week history of cough and sore throat during the winter last year after which her voice became hoarse and strained. It waxes and wanes but is rarely normal. She also reports clearing her throat more frequently. She is a teacher and finds it difficult to teach because of the strain in her voice. Sometimes she also has difficulty yelling at her kids from across the house. On physical exam, her voice is hoarse and mildly strained. She has equal difficulty saying either sentence: "A dog dug a new bone" and "Harry is happy because he has a new horse." There are no breaks in her voice, and there is no stridor.**

1. How difficult would you rate this clinical prompt? Very easy Somewhat easy Neutral

Somewhat difficult Very difficult

Differential Diagnoses:

- 1. Vocal fold nodules
  2. Muscle tension dysphonia
  3. Chronic laryngitis
  4. Reinke's edema (Polypoid corditis)
  5. Vocal fold paralysis or paresis

1. How much do you agree with this list of differential Strongly disagree diagnoses? Somewhat disagree

Neutral Somewhat agree Strongly agree

Treatment:

The most likely diagnosis in this case is vocal fold nodules. Here's a treatment plan for this patient:

- 1. Consults:
     1. Otolaryngology (Ear, Nose, and Throat specialist): for evaluation, confirmation of the diagnosis, and management of vocal fold nodules.
     2. Speech-Language Pathologist: for assessment and voice therapy.
  2. Diagnostic studies:
     1. Laryngoscopy: to visualize the vocal folds and confirm the presence of nodules or other abnormalities.
     2. Videostroboscopy: to assess vocal fold vibration and function, if indicated.
  3. Treatment:
     1. Voice therapy: provided by a speech-language pathologist to improve vocal hygiene, reduce vocal strain, and address compensatory behaviors.
     2. Vocal hygiene: encourage the patient to maintain adequate hydration, avoid caffeine and alcohol, use a humidifier, and minimize throat clearing.
     3. Voice rest: minimize voice use during periods of acute inflammation or after heavy vocal demands.
     4. Vocal warm-ups and cool-downs: to be taught by a speech-language pathologist for use before and after prolonged voice use.
     5. Monitor for improvement with voice therapy; if there is no significant improvement after a few months of therapy, consider re-evaluating the diagnosis and exploring alternative treatments.
  4. Surgical intervention:
     1. In cases where conservative management with voice therapy fails to yield improvement or in severe cases of vocal fold nodules, microlaryngoscopy with excision or laser ablation of the nodules may be considered.

1. For the diagnosis provided by ChatGPT, how much do you Strongly disagree agree with this treatment plan? Somewhat disagree

Neutral Somewhat agree Strongly agree

**Prompt #17:**

**A 55 year-old male underwent a coronary artery bypass graft 5 days ago and presents with a hoarse voice. He has had difficulty with eating, particularly with thin liquids. His voice has not improved since surgery. On physical exam, his voice is hoarse and breathy. When he takes a drink of water, he has overt signs of aspiration. He finds it easier to drink water when he turns his head to the left and tucking his chin.**

1. How difficult would you rate this clinical prompt? Very easy Somewhat easy Neutral

Somewhat difficult Very difficult

Differential Diagnoses:

- 1. Recurrent laryngeal nerve injury or palsy
  2. Vocal cord paralysis
  3. Aspiration pneumonia
  4. Laryngeal edema
  5. Laryngopharyngeal reflux

1. How much do you agree with this list of differential Strongly disagree diagnoses? Somewhat disagree

Neutral Somewhat agree Strongly agree

Treatment:

Based on the most likely diagnosis of recurrent laryngeal nerve injury or palsy following coronary artery bypass graft surgery, I can suggest a general approach for management:

- 1. Consultation: Involve an otolaryngologist (ENT specialist) for a thorough assessment of the patient's symptoms and to confirm the diagnosis.
  2. Diagnostic studies:
     1. Laryngoscopy: A flexible or rigid laryngoscopy can be performed to visualize the vocal cords and assess their mobility. This examination can help confirm vocal cord palsy or other structural abnormalities.
     2. Imaging studies: If necessary, a CT or MRI scan of the neck and chest could be considered to evaluate any potential compressive or infiltrative lesions involving the recurrent laryngeal nerve.
  3. Treatment:
     1. Medical management: Initially, a conservative approach may be taken, which could include voice therapy and swallow therapy with a speech-language pathologist. This approach may help improve vocal function and swallowing techniques to minimize aspiration risks.
     2. Surgical intervention: If the patient's symptoms do not improve or worsen, surgical options such as injection laryngoplasty, medialization thyroplasty, or arytenoid adduction could be considered. These procedures aim to restore vocal cord function and reduce the risk of aspiration.
     3. Monitoring: Regular follow-ups with the otolaryngologist and speech-language pathologist will be essential to monitor the patient's progress and adjust the treatment plan as needed.
  4. Additional considerations:
     1. Aspiration precautions: Educate the patient on safe swallowing techniques (e.g., chin-tuck maneuver) and modify their diet to minimize aspiration risks. This may include thickened liquids, soft foods, and avoiding thin liquids.
     2. Pneumonia prevention: Monitor for signs of aspiration pneumonia, and if it occurs, initiate appropriate antibiotic therapy.

1. For the diagnosis provided by ChatGPT, how much do you Strongly disagree agree with this treatment plan? Somewhat disagree

Neutral Somewhat agree Strongly agree

**Prompt #18:**

**A 3 week-old male presents with a 1 week history of left neck mass. Prenatal history is significant for gestational diabetes. The patient was born post-term via forceps-assisted vaginal delivery. The neck mass has been firm and slowly progressive in size. The patient has otherwise been doing well and has been feeding and gaining weight appropriately. On physical exam, the patient's head is turned slightly to the right. There is a 4 x 3 cm dense mass on the left neck that moves with head turning.**

1. How difficult would you rate this clinical prompt? Very easy Somewhat easy Neutral

Somewhat difficult Very difficult

Differential Diagnoses:

- 1. Congenital muscular torticollis
  2. Sternocleidomastoid tumor (fibromatosis colli)
  3. Lymphatic malformation (cystic hygroma)
  4. Branchial cleft cyst
  5. Hemangioma

1. How much do you agree with this list of differential Strongly disagree diagnoses? Somewhat disagree

Neutral Somewhat agree Strongly agree

Treatment:

The most likely diagnosis in this case is congenital muscular torticollis. Here's a treatment plan for this patient:

- 1. Consults:
     1. Pediatrician: for initial evaluation and management of congenital muscular torticollis.
     2. Physical therapist: for assessment and implementation of stretching and strengthening exercises.
  2. Diagnostic studies:
     1. Ultrasound of the neck: to evaluate the sternocleidomastoid muscle for signs of fibrosis or other abnormalities.
  3. Treatment:
     1. Physical therapy: focused on stretching and strengthening exercises for the affected sternocleidomastoid muscle. The physical therapist will provide guidance on the appropriate techniques and frequency of these exercises.
     2. Parent education: teach parents how to perform the stretching and strengthening exercises, as well as proper positioning techniques to encourage the infant to turn their head to the affected side.
     3. Monitoring: regular follow-up visits with the pediatrician and physical therapist to monitor progress and adjust the treatment plan as needed.
  4. Surgical intervention:
     1. In cases where conservative management with physical therapy fails to yield significant improvement or in severe cases of congenital muscular torticollis, surgical release of the affected sternocleidomastoid muscle may be considered.

1. For the diagnosis provided by ChatGPT, how much do you Strongly disagree agree with this treatment plan? Somewhat disagree

Neutral Somewhat agree Strongly agree

**Prompt #19:**

**A 10 year-old female presents with a painful neck mass over the past 3 days. She states this has happened once before and resolved after a course of oral antibiotics. It is associated with a fever, swelling over the affected area, and pain with swallowing. On physical exam, there is a 3 cm midline tender neck mass that moves when swallowing. It is firm and partially fixed to the overlying skin which has mild erythema. There is bilateral tender cervical lymphadenopathy.**

1. How difficult would you rate this clinical prompt? Very easy Somewhat easy Neutral

Somewhat difficult Very difficult

Differential Diagnoses:

- 1. Acute suppurative thyroiditis
  2. Subacute (De Quervain's) thyroiditis
  3. Lymphadenitis
  4. Thyroglossal duct cyst infection
  5. Abscess

1. How much do you agree with this list of differential Strongly disagree diagnoses? Somewhat disagree

Neutral Somewhat agree Strongly agree

Treatment:

Based on the most likely diagnosis of acute suppurative thyroiditis, I can suggest a general approach for management:

- 1. Consultation: Involve an endocrinologist and/or an otolaryngologist (ENT specialist) for a thorough assessment of the patient's symptoms and to confirm the diagnosis.
  2. Diagnostic studies:
     1. Blood tests: Order a complete blood count (CBC) to assess for leukocytosis, which may indicate infection, and thyroid function tests (TFTs) to evaluate thyroid function.
     2. Imaging studies: Perform an ultrasound of the neck to visualize the thyroid gland and assess for abscess formation or other structural abnormalities. In some cases, a contrast-enhanced CT scan may be necessary for further evaluation.
     3. Fine-needle aspiration (FNA): If an abscess is suspected, an FNA can be performed to obtain a sample for culture and sensitivity testing, which can guide antibiotic therapy.
  3. Treatment:
     1. Medical management: Initiate empirical antibiotic therapy, typically with intravenous broad-spectrum antibiotics, until culture and sensitivity results are available. Antibiotics may then be adjusted based on the results. Continue antibiotic treatment for 2-3 weeks or as recommended by the specialist.
     2. Surgical intervention: If an abscess is confirmed and does not respond to antibiotic therapy or if the patient's condition worsens, incision and drainage or surgical intervention may be necessary. This may include drainage of the abscess or, in rare cases, partial or total thyroidectomy.
     3. Monitoring: Regular follow-ups with the endocrinologist and otolaryngologist will be essential to monitor the patient's progress and adjust the treatment plan as needed.
  4. Additional considerations:
     1. Pain management: Administer analgesics as needed to manage the patient's pain.
     2. Fever management: Antipyretics can be used to manage fever.

1. For the diagnosis provided by ChatGPT, how much do you Strongly disagree agree with this treatment plan? Somewhat disagree

Neutral Somewhat agree Strongly agree

**Prompt #20:**

**A 41 year-old female presents with 1 week history of left otalgia. It has been progressively worse since it started and has spread to her left eye today. She also endorses double vision and hearing loss but denies tinnitus, otorrhea and vertigo. She has insulin-dependent diabetes mellitus. On physical exam, she appears fatigued. She has a mild left eye abduction deficit. She has normal pinna and external auditory canals bilaterally. There is a milky left middle ear effusion, but her tympanic membrane appears relatively normal. She has a temperature of 101.4F. His tuning fork exam lateralizes to the left, and air conduction is greater than bone conduction on the right but bone conduction is greater than air conduction on the left. Her facial nerve is intact.**

1. How difficult would you rate this clinical prompt? Very easy Somewhat easy Neutral

Somewhat difficult Very difficult

Differential Diagnoses:

- 1. Acute otitis media with complications
  2. Mastoiditis
  3. Gradenigo's syndrome
  4. Cholesteatoma with complications
  5. Temporal bone osteomyelitis

1. How much do you agree with this list of differential Strongly disagree diagnoses? Somewhat disagree

Neutral Somewhat agree Strongly agree

Treatment:

The most likely diagnosis in this case is acute otitis media with complications. Here's a treatment plan for this patient:

- 1. Consults:
     1. Otolaryngology (Ear, Nose, and Throat specialist): for evaluation, confirmation of the diagnosis, and management of acute otitis media and possible complications.
  2. Diagnostic studies:
     1. Audiometry: to assess the extent of hearing loss.
     2. Computed tomography (CT) scan of the temporal bone: to evaluate the middle ear and mastoid structures for complications such as mastoiditis or cholesteatoma.
     3. Blood tests: to evaluate for infection and inflammation (complete blood count, C-reactive protein, and erythrocyte sedimentation rate).
  3. Treatment:
     1. Antibiotics: empiric broad-spectrum antibiotics should be initiated while awaiting culture results to target the most likely pathogens (e.g., amoxicillin-clavulanate or ceftriaxone).
     2. Analgesics: pain management with acetaminophen or ibuprofen.
     3. Antipyretics: to manage fever.
     4. Close monitoring: for clinical improvement and to assess for possible complications or the need for surgical intervention.
  4. Surgical intervention:
     1. If complications such as mastoiditis or cholesteatoma are identified, the patient may require surgical intervention, such as a myringotomy with or without tympanostomy tube placement, or a mastoidectomy.

1. For the diagnosis provided by ChatGPT, how much do you Strongly disagree agree with this treatment plan? Somewhat disagree

Neutral Somewhat agree Strongly agree
