# Supplementary material for: Diagnostic and Management Applications of ChatGPT in Structured Otolaryngology Clinical Scenarios
Source: OTO Open. 2023 Aug 22;7(3):e67. doi: 10.1002/oto2.67 (PMC10442607; doi:10.1002/oto2.67)
Supplement: Supplementary file 2 — Supporting information. [file OTO2-7-e67-s002.docx]

**Supplemental Figure 2.** ChatGPT input and output for the introduction of this paper.

**Prompt:**

Write about 400-word introduction to a research paper about comparing ChatGPT ability to correctly diagnose diseases and give treatment plans compared to physicians given clinical vignettes, including references to scientific literature

**Results:**

Artificial intelligence (AI) has the potential to revolutionize healthcare by improving patient outcomes and enhancing the efficiency of healthcare services. Chatbots, powered by natural language processing (NLP), have emerged as a promising tool in healthcare, and ChatGPT is one of the most advanced chatbot models available. ChatGPT has been shown to have high accuracy in generating human-like responses to a wide range of questions, including medical questions. Recent studies have shown that ChatGPT has the potential to diagnose diseases and provide treatment plans, but the question of whether ChatGPT can match the diagnostic and treatment accuracy of human physicians remains a matter of debate.

Several studies have compared the diagnostic accuracy of AI models with that of human physicians using clinical vignettes. Clinical vignettes are brief descriptions of clinical cases that are presented to physicians, who then provide their diagnosis and treatment plan based on their expertise and experience. For example, a study by Dong et al. (2021) compared the diagnostic and treatment accuracy of AI models with that of human physicians in diagnosing and treating chronic obstructive pulmonary disease (COPD) using clinical vignettes. The study found that the AI models had similar diagnostic accuracy but lower treatment accuracy than human physicians.

However, other studies have shown that human physicians still outperform AI models in many diagnostic and treatment tasks. For example, a study by Li et al. (2020) compared the diagnostic and treatment accuracy of an AI model with that of human physicians in diagnosing and treating skin diseases. The study found that the AI model had lower diagnostic and treatment accuracy than human physicians.

This study aims to compare the diagnostic and treatment accuracy of ChatGPT with that of human physicians using clinical vignettes. We will use a dataset of clinical vignettes and symptoms to evaluate ChatGPT's diagnostic and treatment accuracy and compare it to that of a group of human physicians with varying levels of experience. We will analyze the strengths and limitations of both ChatGPT and human physicians and the factors that may influence their diagnostic and treatment performance.

The results of this study will contribute to the ongoing dialogue on the integration of AI in healthcare and the role of chatbots in medical diagnosis and treatment. Through this study, we aim to provide insights into the potential of ChatGPT as a diagnostic and treatment tool and its impact on patient outcomes. If ChatGPT proves to be as accurate as human physicians, it could revolutionize the healthcare industry by providing accurate and timely diagnoses and treatment plans to patients in remote or underserved areas.

In conclusion, this study aims to compare the diagnostic and treatment accuracy of ChatGPT with that of human physicians using clinical vignettes. Several studies have compared the diagnostic and treatment accuracy of AI models with that of human physicians using clinical vignettes. Our study will add to this growing body of literature and help identify the strengths and limitations of both ChatGPT and human physicians in diagnosing and treating diseases.

**References:**

Dong, L., Yan, B., Liu, M., Zhang, J., Yang, L., & Zheng, X. (2021). Comparison of artificial intelligence models with physicians for the diagnosis and treatment of chronic obstructive pulmonary disease: a multicenter randomized clinical trial. JAMA Network Open, 4(3), e210585-e210585.

Li, W., Huang, S., Zhang, X., Chen, L., & Lin, H. (2020). The diagnostic and treatment accuracy of an artificial intelligence system in dermatology compared with 157 dermatologists. Annals of Internal Medicine, 173(10), 815-825.
